# Progress Toward High Power Output in Thermionic Energy Converters

**DOI:** 10.1002/advs.202003812

**Published:** 2021-03-03

**Authors:** Matthew F. Campbell, Thomas J. Celenza, Felix Schmitt, Jared W. Schwede, Igor Bargatin

**Affiliations:** ^1^ Department of Mechanical Engineering and Applied Mechanics University of Pennsylvania Philadelphia PA 19104 USA; ^2^ Spark Thermionics, Inc. Emeryville CA 94608 USA

**Keywords:** efficiency, heat transfer, power density, thermionic energy conversion

## Abstract

Thermionic energy converters are solid‐state heat engines that have the potential to produce electricity with efficiencies of over 30% and area‐specific power densities of 100 Wcm^−2^. Despite this prospect, no prototypes reported in the literature have achieved true efficiencies close to this target, and many of the most recent investigations report power densities on the order of mWcm^−2^ or less. These discrepancies stem in part from the low‐temperature (<1300 K) test conditions used to evaluate these devices, the large vacuum gap distances (25–100 µm) employed by these devices, and material challenges related to these devices' electrodes. This review will argue that, for feasible electrode work functions available today, efficient performance requires generating output power densities of >1 Wcm^−2^ and employing emitter temperatures of 1300 K or higher. With this result in mind, this review provides an overview of historical and current design architectures and comments on their capacity to realize the efficiency and power potential of thermionic energy converters. Also emphasized is the importance of using standardized efficiency metrics to report thermionic energy converter performance data.

## Graphical abstract

Thermionic energy converters transform heat into electricity using no moving parts. This review highlights recent developments that have the potential to achieve high power density outputs at high efficiencies for commercial operation. Also emphasized are two standardized efficiency metrics to evaluate converter performance.

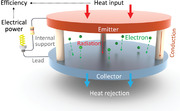

## 1 Introduction

Thermionic energy converters (TECs) are heat engines that convert very high‐temperature heat directly into electricity by driving electrons across a vacuum gap, allowing for high efficiencies without any moving parts.^[^ ^1^ ^]^ Operating at high temperatures allows TECs to accept heat directly from a variety of sources^[^ ^2^ ^]^ such as hydrocarbon combustion,^[^ ^3^ ^]^ concentrated sunlight,^[^ ^4^ ^]^ or nuclear generation processes,^[^ ^5^ ^]^ and, in many cases, to reject unused heat to the environment without large heat exchangers. Additionally, the lack of moving parts can give TECs inherently long lifetimes with little associated maintenance and lets them avoid some irreversible loss mechanisms such as friction and turbulence. Finally, TECs have relatively small sizes that can provide high specific power outputs up to *P* ≈ 100 Wcm^−2^.

A significant amount of research on TECs has occurred, as summarized in numerous review papers,^[^ ^3^, ^4^, ^6^, ^7^, ^8^, ^9^, ^10^, ^11^, ^12^, ^13^, ^14^, ^15^, ^16^, ^17^, ^18^, ^19^, ^20^ ^]^ including several published within the last few years that highlight multiple new concepts and approaches.^[^ ^21^, ^22^, ^23^, ^24^, ^25^, ^26^, ^27^ ^]^ Despite the recent interest directed toward TECs, few modern TECs have demonstrated efficiencies close to the technology's potential or have achieved power densities of more than tens of mWcm^−2^. Therefore, a major purpose in writing this review is to evaluate the ability of recent developments to enable significant power density outputs (*P* > 1 Wcm^−2^), which, as we explain below, are typically also necessary for heat‐to‐electricity conversion efficiencies of practical interest (η>10%).

The ability of TECs to generate high power density stems from their unique architecture, which in turn distinguishes them from similar direct energy conversion technologies such as thermoelectric converters and thermophotovoltaic devices (TPVs). Unlike thermoelectrics, which employ a semiconductor between the hot and cold sides of the device, TECs employ a thin vacuum gap, which allows them to operate at very high temperatures without excessive parasitic internal heat loss.^[^ ^28^, ^29^, ^30^, ^31^ ^]^ Moreover, unlike TPVs, which convert the thermal radiation generated by a hot surface into electricity using a photovoltaic cell, TECs directly deliver electric current as electrons emerge from the emitter, thereby offering the possibility for much higher power densities (up to 10–100 Wcm^−2^) within smaller form factors.^[^ ^32^, ^33^ ^]^ Typical benchmark efficiencies (see Equation (6) later in this article) for practical thermionic, thermoelectric, and thermophotovoltaic converters are all of order 10%, though some examples of high efficiency architectures have been reported.^[^ ^34^, ^35^ ^]^ We will examine various efficiency metrics for thermionic converters later in this review.

## 2 Technological Overview

### 2.1 Governing Equations

**Figure 1:**
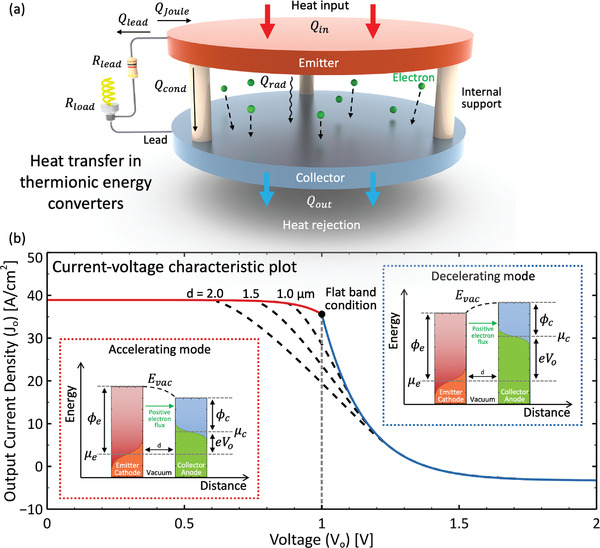
a) Schematic diagram of a diode thermionic energy converter. Heat transferred into the emitter $Q_{\text{in}}$ is transferred out by the flow of thermionic electrons to the collector $Q_{\text{therm}}$ (shown by dashed arrows but not labeled), radiation to the collector $Q_{\text{rad}}$, and conduction through the lead wires $Q_{\text{lead}}$. A portion of the Joule heating in the lead wires contributes $Q_{\text{Joule}}$ to the emitter, while the rest (not shown) flows to the collector. If internal supports (shown as white columns) are used to separate the emitter and collector, conduction $Q_{\text{cond}}$ occurs through them as well. Also, if residual vapors (such as cesium) are present in the interelectrode space, gas conduction and convection $Q_{\text{vapor}}$ take place. Other heat losses, such as conduction through structural components, are not shown. The total heat transferred away from the collector is represented by $Q_{\text{out}}$. The electrical resistance of the lead wires is $R_{\text{lead}}$ and the electrical load $R_{\text{load}}$ is depicted as a light bulb. b) Current–voltage characteristic plot for a generic TEC. The solid lines are calculated by neglecting space charge, and the black dashed lines show calculations that include space charge effects at electrode gap distances of d=1.0, 1.5, and 2.0 µm. The insets show electron motive diagrams for operation in accelerating mode and decelerating mode, where the curved black dashed lines represent the position‐dependent vacuum energy level $E_{\text{vac}}$ and the gap width d is defined. The orange and green shaded areas in the emitter and collector are the respective electron Fermi–Dirac energy distributions. Also, $J_{o}$ is the output current density, $V_{o}$ is the output voltage, e is the electron charge, and $\mu_{e}$ and $\mu_{c}$ are the Fermi levels of the emitter and collector, respectively. This plot was produced for $A_{e}=A_{c}=120$ A cm ^−2^ K^−2^, $\phi_{e}=2.5$ eV, $\phi_{c}=1.5$ eV, $T_{e}=1800$ K, and $T_{c}=1000$ K. The flat‐band condition occurs at $V_{\text{fbc}}=\left(\phi_{e}-\phi_{c}\right)\;/e=1$ V, and the space charge calculations were performed according to equations available in Hatsopoulos and Gyftopoulos.^[^ ^13^, ^37^ ^]^

A simple TEC is shown in **Figure** **1**a. It consists of a hot emitter electrode (cathode) separated by a thin vacuum or plasma gap of distance d [µm] from a relatively cold collector electrode (anode). A fraction of the electrons in the hot emitter have sufficient kinetic energy to spontaneously emerge into the vacuum gap in a process called thermionic emission. These electrons traverse the gap, enter the collector, and return to the emitter through the lead wires and load to complete the electric circuit. In the figure, the gap is maintained by internal spacer columns but can alternatively be sustained by external supports. As summarized by steady‐state energy conservation in Equation (1), heat input delivered to the emitter at flux $Q_{\text{in}}$ [Wcm^−2^] is transferred by means of thermionic electron flow ($Q_{\text{therm}}$), conduction through internal supports ($Q_{\text{cond}}$), gas conduction or convection through residual vapors in the gap ($Q_{\text{vapor}}$), and radiation ($Q_{\text{rad}}$) from the emitter to the collector.

(1) $$Q_{\text{in}}=Q_{\text{therm}}+Q_{\text{cond}}+Q_{\text{vapor}}+Q_{\text{rad}}+Q_{\text{lead}}-Q_{\text{Joule}}$$

Some heat is also conducted away from the emitter through the lead wire ($Q_{\text{lead}}$) owing to its finite thermal resistance, which is typically related to its electrical resistance $R_{\text{lead}}$ [Ω] by the Wiedemann–Franz law. However, Joule heating in the lead wires contributes some heat to the emitter ($Q_{\text{Joule}}$). The radiative, lead, and Joule heat components are fundamental, meaning that they cannot be avoided even in theory (unless, for instance, the lead is replaced by a thermoelectric leg^[^ ^36^ ^]^). The conductive losses are not fundamental but in practice nearly always occur to some degree either through the gap‐maintaining spacers or through external supports. In contrast, the convective losses only occur in the presence of residual vapors (often cesium) within the gap. Heat $Q_{\text{out}}$ is ultimately carried away from the collector, typically by a finned heat exchanger that rejects heat to the environment. We will first examine the thermionic heat transfer below and will treat the other heat transfer mechanisms subsequently.

The Richardson‐Dushmann equation describes the electron current density J [Acm^−2^] produced by an electrode with temperature T [K] and electron emission energy barrier $E_{b}$ [eV].^[^ ^38^, ^39^ ^]^

(2) $$J=AT^{2}\exp\;\left(\frac{-E_{b}}{k_{B}T}\right)$$

Here, A [Acm^−2^ K^−2^] is the electrode's Richardson constant and $k_{B}$ is the Boltzmann constant [eV K^−1^]. The theoretical value of A can be calculated as $A=\frac{4\pi em_{\text{el}}k_{B}^{2}}{h^{3}}\approx 120$ Acm^−2^ K^−2^ (where $e\approx 1.6\times 10^{-19}$ C is the electron charge, $m_{\text{el}}$ [kg] is the electron mass, and h [eVs] is Planck's constant), although in practice A‐values depend on the material and are often much smaller. We note that both the emitter and the collector electrodes can emit electrons, implying that the net output current density produced by a TEC is given by $J_{o}=J_{e}-J_{c}$ where here and henceforth subscripts “e” and “c” denote the emitter and collector, respectively.

Figure 1b shows a current–voltage characteristic plot for a generic TEC. We will first discuss the red and blue solid lines, which were calculated without considering any non‐ideal barrier (e.g., the space charge effect) within the gap. The left side of the plot is known as the *accelerating region*, in which the output current density $J_{o}$ remains roughly constant (absent substantial Schottky barrier lowering) with increasing output voltage $V_{o}$ [V] until the flat‐band condition $V_{o}=V_{\text{fbc}}=\left(\phi_{e}-\phi_{c}\right)/e$, where $\phi_{e}$ and $\phi_{c}$ [eV] are the work functions of the emitter and collector, respectively (note that this equation requires a unit conversion of $1\;\text{eV}\approx 1.6\times 10^{-19}\;V\cdot C=1.6\times 10^{-19}\;J$). At higher output voltages, in the so‐called *decelerating region*, $J_{o}$ drops exponentially because it is controlled by the population of electrons with sufficient Boltzmann‐distributed energy to escape into the vacuum and overcome the additional decelerating electric field in the interelectrode gap.^[^ ^40^ ^]^ The net current can become negative at high voltages because a small amount of reverse current from the collector $J_{c}$ overcomes that originating from the emitter $J_{e}$.

The insets of Figure 1b show the electrons' potential energy. The left diagram corresponds to the accelerating region; it reveals that, neglecting any nonideal barriers, electrons in the emitter with energy $E-\mu_{e}$ greater than or equal to the energy barrier $E_{b}=\phi_{e}$ may travel through the gap and enter the collector (here, $\mu_{e}$ [eV] is the Fermi level of the emitter). Simultaneously, electrons in the collector with $E-\mu_{c}>E_{b}=\phi_{e}-eV_{o}$ may travel into the emitter ($\mu_{c}$ is the collector Fermi level). In decelerating mode (the right diagram), electrons in the emitter with $E-\mu_{e}>E_{b}=\phi_{c}+eV_{o}$ may travel into the collector, and electrons in the collector with $E-\mu_{c}>E_{b}=\phi_{c}$ may travel back into the emitter.

The vacuum level $E_{\text{vac}}$ is position‐dependent, and in practical operation may have a maximum between the emitter and collector. This additional energy barrier is caused by the presence of electrons within the gap that create a negative electric field that opposes the flow of electrons leaving the electrodes, which is known as the *space charge effect*. The additional space charge energy barrier modifies the current–voltage characteristic lines, and the extent of this alteration is a function of the interelectrode distance d. Figure 1b provides current–voltage profiles that account for space charge at gap distances of d=1.0, 1.5, and 2.0 µm, calculated using methods detailed elsewhere^[^ ^13^, ^37^, ^41^ ^]^ and shown as black dashed lines. We will discuss strategies to mitigate the impact of space charge later in this article. Note that space charge effects also occur in other electronic devices. Under the assumption that electrons are emitted with zero initial velocity, the physics can be described by the simple Child–Langmuir law, as summarized by Zhang et al.^[^ ^42^ ^]^ However, thermionic energy converters consider the case in which emitted electrons possess a thermal energy distribution, and in practice these thermal effects are important during operation.

A historically important figure of merit for thermionic converters is the *back voltage* $V_{b}$ [V], sometimes referred to as the *barrier index*.^[^ ^14^, ^18^ ^]^ In the accelerating region (left inset in Figure 1b) or during space–charge limited operation, $V_{b}$ can be calculated according to Equations (3) and (4), in which $V_{g}$ [V] is the gap voltage, necessary to overcome space charge within the interelectrode space.

(3) $$\begin{array}{cc} V_{b} & =V_{g}+\frac{\phi_{c}}{e}=\frac{\phi_{e}}{e}-V_{o} \end{array}$$

(4) $$\begin{array}{cc} V_{g} & =\frac{\phi_{e}}{e}-\frac{\phi_{c}}{e}-V_{o} \end{array}$$

The back voltage is often evaluated at the maximum power point; lower values of $V_{b}$ correspond to improved TEC performance because they yield higher output voltages.

The electrical power density P [Wcm^−2^] produced by a TEC is given by

(5) $$P=J_{o}\left(V_{o}-J_{o}S_{e}R_{\text{lead}}\right)$$

where $S_{e}$ [cm^2^] is the electron‐emitting surface area of the emitter and $J_{o}S_{e}R_{\text{lead}}$ corresponds to the voltage drop associated with electrical resistance in the lead wires. Naturally, the power density changes with the current density and voltage; the current density and voltage associated with the maximum power density point are often given the subscript “mpp”. Note that the current and voltage corresponding to the maximum power point do not necessarily correspond to those at the point of maximum efficiency. The TEC's efficiency, η  [%], is the ratio of the power density P to the total input heat flux to the emitter $Q_{\text{in}}$.

(6) $$\eta =\frac{P}{Q_{\text{in}}}\times 100\%$$

Since this metric reflects the essential TEC diode's performance, we will refer to this as the *core* *efficiency*. Importantly, our definition differs from the *ideal* *efficiency* introduced by Hatsopoulos and Gyftopoulos,^[^ ^13^ ^]^ because it includes the impact of conduction through support structures or residual vapor in the interelectrode gap. We will discuss other efficiency metrics in Section 6.1.

The fundamental heat transfer terms necessary to obtain $Q_{\text{in}}$ [Wcm^−2^] using Equation (1) can be computed according to

(7) $$\begin{array}{cc} Q_{\text{therm}} & =\left(\frac{\phi_{e}}{e}\right)\;\left(J_{e}-J_{c}\right)+\left(\frac{2k_{B}}{e}\right)\;\left(J_{e}T_{e}-J_{c}T_{c}\right) \end{array}$$

(8) $$\begin{array}{cc} Q_{\text{rad}} & =\sigma \varepsilon_{\text{eff}}\;\left(T_{e}^{4}-T_{c}^{4}\right) \end{array}$$

(9) $$\begin{array}{cc} Q_{\text{lead}} & =\frac{L}{2S_{e}R_{\text{lead}}}\;\left(T_{e}^{2}-T_{c}^{2}\right) \end{array}$$

(10) $$\begin{array}{cc} Q_{\text{Joule}} & =\frac{1}{2}S_{e}R_{\text{lead}}\;{\left(J_{e}-J_{c}\right)}^{2} \end{array}$$

where σ [Wcm^−2^K^−4^] is the Stefan–Boltzmann constant, $\varepsilon_{\text{eff}}={\left(\frac{1}{\varepsilon_{e}}+\frac{1}{\varepsilon_{c}}-1\right)}^{-1}$ is the effective interelectrode emissivity derived from the emitter and collector emissivity values, $\varepsilon_{e}$ and $\varepsilon_{c}$, respectively (see Goodman^[^ ^43^ ^]^), and L [WΩK^−2^] is the Lorenz number.^[^ ^13^, ^44^ ^]^ Equation (7) can be obtained by considering the electron fluxes to and from the emitter as well as the energy flux associated with electrons in the lead wires. Equation (9) can be derived by inserting the Wiedemann–Franz law at an average electrode temperature $\frac{1}{2}\;\left(T_{e}+T_{c}\right)$ into the Fourier heat conduction law for heat transfer along the leads connecting the emitter and collector. In Equation (10), we assume for simplicity that one half of the total energy generated through Joule heating in the leads flows back to the emitter, although the actual proportion depends on how the resistivity and the thermal conductivity depend on temperature, as well as the exact geometry of the leads.

The conductive heat transfer, which is not fundamental to TECs but is often present in practical vacuum‐encapsulated structures as well as through structures that separate the electrodes, is given in Equation (11).

(11) $$Q_{\text{cond}}=\frac{1}{\varrho_{s}}\;\left(T_{e}-T_{c}\right)$$

Here $\varrho_{s}$ [cm^2^K W^−1^] is the total thermal resistance of the interelectrode spacer or any other support structures that conduct heat parasitically from the emitter to the collector.

Finally, converters whose electrodes have an alkali metal coating, most commonly cesium or barium, experience heat transfer due to convection and conduction by residual vapors $Q_{\text{vapor}}$.^[^ ^13^, ^45^, ^46^ ^]^ The following equations can be used to determine the heat flux due to cesium vapor in the free molecular conduction regime,^[^ ^47^ ^]^ where the cesium atoms' mean free path λ is much greater than the interelectrode gap distance d, or equivalently, where the Knudsen number $Kn=\frac{\lambda }{d}\gg 1$.

(12) $$\begin{array}{cc} Q_{\text{vapor}} & =\frac{a_{\mathrm{Cs}}}{2-a_{\mathrm{Cs}}}\;P_{\mathrm{Cs}}\;{\left(\frac{8k_{B}}{\pi m_{\mathrm{Cs}}}\right)}^{1/2}\;\left(\sqrt{T_{e}}-\sqrt{T_{c}}\right) \end{array}$$

(13) $$\begin{array}{cc} P_{\mathrm{Cs}} & =3.2664\times 10^{10}\frac{1}{\sqrt{T_{r}}}\exp\;\left(\frac{-8910}{T_{r}}\right) \end{array}$$

Note that, as written, Equation (12) yields $Q_{\text{vapor}}$ values with units of Wm^−2^ (rather than Wcm^−2^), Equation (13) provides values for the cesium pressure $P_{\mathrm{Cs}}$ in pascals, $a_{\mathrm{Cs}}=0.8$ is the accommodation coefficient of cesium,^[^ ^47^ ^]^ $m_{\mathrm{Cs}}$ [kg] is the mass of a cesium atom, and $T_{r}$ [K] is the cesium reservoir temperature (often $500<T_{r}<700$ K). For higher cesium pressures or larger gap distances in the transition flow regime (where Kn≈1), Equation (12) serves as an upper bound, and actual values can be calculated using methods presented elsewhere.^[^ ^13^, ^47^ ^]^

### 2.2 Link Between Output Power Density, Core Efficiency, and Emitter Temperature

**Figure 2:**
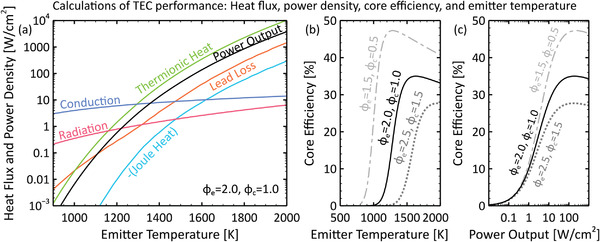
a) Thermionic ($Q_{\text{therm}}$), gap spacer conductive ($Q_{\text{cond}}$), radiative ($Q_{\text{rad}}$), and lead conductive ($Q_{\text{lead}}$) heat transfer fluxes away from the emitter, and output power density P as a function of emitter temperature $T_{e}$. The contribution of the Joule heating in the leads $Q_{\text{Joule}}$ (directed toward the emitter) is also provided. Conditions: $T_{c}=600$ K, $\phi_{e}=2.0$ eV, $\phi_{c}=1.0$ eV, $A_{e}=A_{c}=120$ Acm^−2^K^−2^, $\varepsilon_{e}=0.2$, $\varepsilon_{c}=0.1$, $\varrho_{s}=100$ cm^2^K W^−1^, and $S_{e}=1$ cm^2^. b) Core efficiency η as a function of emitter temperature for three electrode work function combinations. Solid line: $\phi_{e}=2.0$ eV, $\phi_{c}=1.0$ eV, $T_{c}=600$ K; dash‐dot line: $\phi_{e}=1.5$ eV, $\phi_{c}=0.5$ eV, $T_{c}=300$ K; dotted line: $\phi_{e}=2.5$ eV, $\phi_{c}=1.5$ eV, $T_{c}=900$ K. c) TEC core efficiency as a function of output power density (conditions identical to (b)). To create these graphs, we iteratively determined the output voltage $V_{o}$ and lead resistance $R_{\text{lead}}$ that maximized the core efficiency η at each emitter temperature $T_{e}$. We did not consider space charge effects, structural heat transfer losses, or heat transfer through residual vapor in the gap. For results with optimized collector temperatures, see Figure 10.

**Figure** **2** provides a comparison of the net thermionic $Q_{\text{therm}}$, conductive $Q_{\text{cond}}$ (through a gap spacer or internal support structure), radiative $Q_{\text{rad}}$, and lead loss $Q_{\text{lead}}$ heat transfer fluxes (Equations (7)–(9) and (11)) away from the emitter for a generic vacuum micron‐gap TEC. The part of the Joule heating in the leads that is directed toward the emitter $Q_{\text{Joule}}$ (Equation (10)) is provided as well, and the output electrical power density P, given by Equation (5), is shown for comparison. To model the conduction, we chose a spacer thermal resistivity value of $\varrho_{s}=100$ cm^2^K W^−1^, which is representative of the best values in recent publications on interelectrode spacers for thermionic devices.^[^ ^48^ ^]^ We have shown a generic best‐case operational scenario where the space charge effect, conduction to external elements, and heat transfer due to cesium vapor in the gap are negligible. For simplicity, we have also selected collector temperatures based on the collector work function (i.e., $T_{c}\;\text{[K]}=600\times \phi_{c}\;\text{[eV]}$), as well as a constant difference in emitter and collector work function of $\phi_{e}-\phi_{c}=1$ eV. These simplifications somewhat reduce the calculated efficiencies, especially at high emitter temperatures, but broadly similar results can be obtained using optimized collector temperatures and work functions, as shown by Hatsopoulos and Gyftopoulos^[^ ^13^ ^]^ and Figure 10 later in this article.

In Figure 2a, where $\phi_{e}=2$ eV and $\phi_{c}=1$ eV, thermionic heat transfer is dominated by conductive and radiative losses unless temperatures exceed about $T_{e}\approx 1300$ K. Therefore, according to Equations (1), (5), and (6), the core efficiency remains low (η<10%) until the thermionic, conductive, and radiative heat fluxes achieve similar orders of magnitude (≈1 Wcm^−2^). This threshold behavior is reflected in Figure 2b, which shows the TEC core efficiency increasing sharply near $T_{e}\approx 1300$ K for the ($\phi_{e}=2$ eV, $\phi_{c}=1$ eV) calculation. Figure 2b also provides core efficiency *versus* emitter temperature trends for two other work function pairs, namely ($\phi_{e}=1.5$ eV, $\phi_{c}=0.5$ eV) and ($\phi_{e}=2.5$ eV, $\phi_{c}=1.5$ eV). The core efficiency of the ($\phi_{e}=1.5$ eV, $\phi_{c}=0.5$ eV) pair is seen to increase at a lower temperature of $T_{e}\approx 1000$ K. However, this work function combination is very optimistic; as depicted later in Figure 4, few examples exist of collector work functions with $\phi_{c}<1$ eV, and even fewer can sustain large electric currents. Thus, the values provided by the ($\phi_{e}=2$ eV, $\phi_{c}=1$ eV) and ($\phi_{e}=2.5$ eV, $\phi_{c}=1.5$ eV) calculations, which show efficiency values increasing near $T_{e}\approx 1300$ K and $T_{e}\approx 1500$ K, respectively, are more realistic. If conduction through electrode supports were suppressed completely, the temperatures at which the efficiency records in Figure 2b increase would be reduced by only about 120 K, revealing that the fundamental heat transfer modes alone still constrain TEC performance. Clearly, given existing limitations in terms of available materials with appropriate work functions, TECs must be operated at high temperatures in order to achieve reasonable energy conversion efficiencies (η>10%).

Figure 2c highlights the important relationship between core efficiency and output power density. Efficiency values in all three work function pair calculations remain low until power density values exceed a rough threshold of about P≈1 Wcm^−2^, at which point they rise above η≈10%. In addition, the plot suggests that high output efficiencies (η>35%) can be achieved only if power densities of P≈100 Wcm^−2^ can be realized. Likewise, core efficiencies near η≈25% will be impossible to achieve unless output power densities can be made greater than P≈10 Wcm^−2^, thereby requiring input heat fluxes of order $Q_{\text{in}}\approx 40$ Wcm^−2^. At lower power densities, conduction and radiation losses dominate the overall energy balance, resulting in much lower efficiency values. Again, if interelectrode conduction was eliminated, the efficiency records in Figure 2c would increase at power densities roughly one order of magnitude smaller; thus, increasing the thermal resistance $\varrho_{s}$ of internal structural components is an important area of research (see also Figure 11). Nevertheless, Figure 2 emphasizes that, for practical thermionic devices with realistic thermal conduction, high power output and high efficiency go hand‐in‐hand, and moreover that these are inextricably linked to high‐temperature operation, given the work functions of currently available electrode materials.

**Figure 3:**
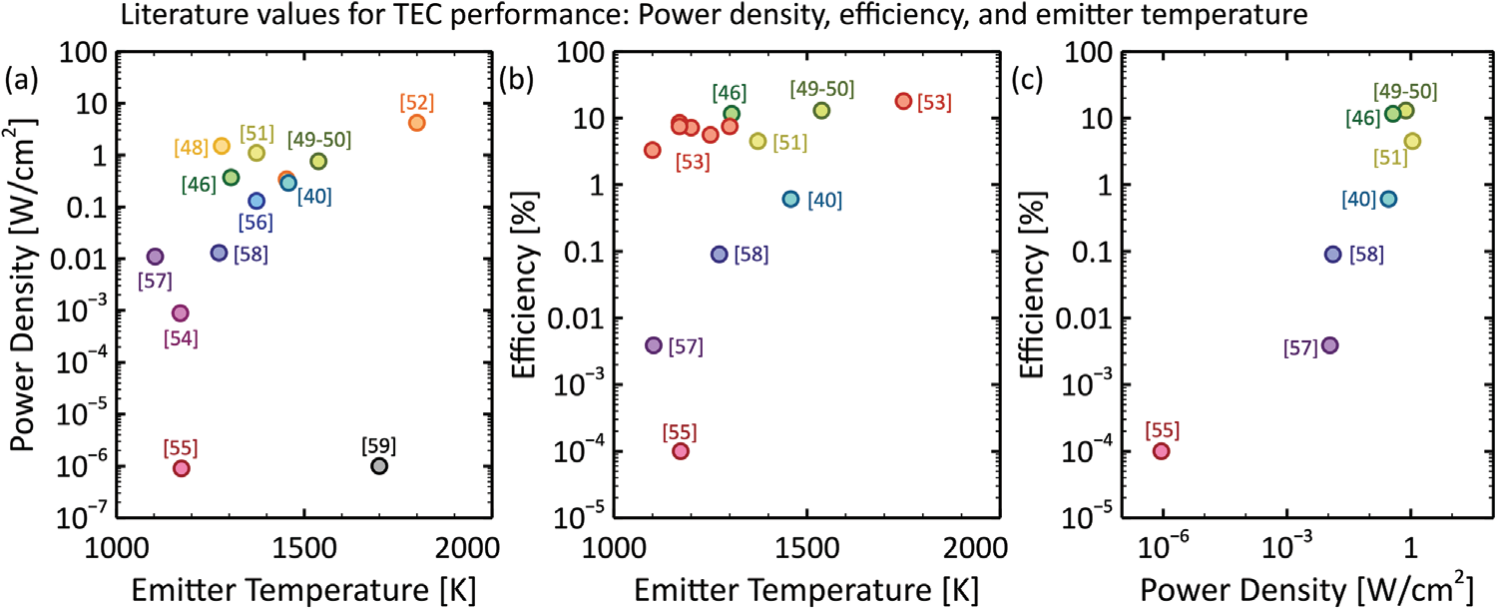
Reported power density, efficiency, and emitter temperature values of several non‐plasma‐based TECs in the literature.^[^ ^40^, ^46^, ^48^, ^49^, ^50^, ^51^, ^52^, ^53^, ^54^, ^55^, ^56^, ^57^, ^58^, ^59^ ^]^ The numbers in brackets refer to the references at the end of this article. In panel (a), the TEC constructed by Bellucci  et al.^[^ ^59^ ^]^ was a proof‐of‐concept device whose high work function electrodes limited its current density (note that another paper by Bellucci  et al.^[^ ^60^ ^]^ describes a different early prototype that falls below the range of this plot). Also in panel (a), the point behind Littau  et al.^[^ ^40^ ^]^ is associated with Dick et al.^[^ ^52^ ^]^ In panel (b), several points are associated with Fitzpatrick et al.^[^ ^53^ ^]^

Finally, **Figure** **3** shows experimental power density, efficiency, and emitter temperature values for several TECs in the literature.^[^ ^40^, ^46^, ^48^, ^49^, ^50^, ^51^, ^52^, ^53^, ^54^, ^55^, ^56^, ^57^, ^58^, ^59^ ^]^ We have not included TECs that use an intragap plasma for space charge mitigation (discussed later), but such devices often achieve power densities above ≈1 Wcm^−2^ and efficiencies of ≈10%.^[^ ^9^, ^12^, ^14^, ^15^, ^61^, ^62^ ^]^ Due to ambiguity about the efficiency calculation method in each of these references, these are not necessarily the same as the *core* *efficiency*, defined above in Equation (6). We will propose a method for reporting standardized efficiencies in Section 6.1. Despite these points collectively representing a wide range of gap distances, work function values, and TEC architectures, they show that power density generally increases with emitter temperature (Figure 3a), efficiency generally increases with emitter temperature (Figure 3b), and efficiency generally increases with power density (Figure 3c). The highest efficiencies of slightly over 10% were reported for output power density values of ≈1 Wcm^−2^, in agreement with Figure 2.

### 2.3 Design Considerations

There are several factors that should be recognized when designing practical TECs, the most important of which is the space charge effect mentioned above. Three primary strategies to reduce this burden have been 1) to neutralize the space charge using plasma within the gap (usually using cesium ions), 2) to employ a third positively charged electrode, sometimes called a *gate* or *grid*, that produces an electron‐accelerating force, or 3) to minimize the gap distance (often to d<10 µm) to reduce the total number of electrons in the gap.^[^ ^44^ ^]^ TECs using a plasma are often associated with a 30–50% reduction in efficiency due to the power required to maintain the plasma and because the plasma ions interfere with the electron flow.^[^ ^17^ ^]^ TECs using a gate also have an associated efficiency penalty because the gate intercepts a fraction of the electrons, thereby requiring a significant power input to maintain the gate at an appropriate voltage.^[^ ^56^ ^]^ Finally, while micron‐scale gap sizes (d<10 µm) are beneficial for overcoming space charge, electrode distances should be designed larger than a minimum threshold (d>0.5 µm), below which parasitic losses due to near‐field radiative heat transfer effects become prominent.^[^ ^44^, ^63^, ^64^ ^]^

**Figure 4:**
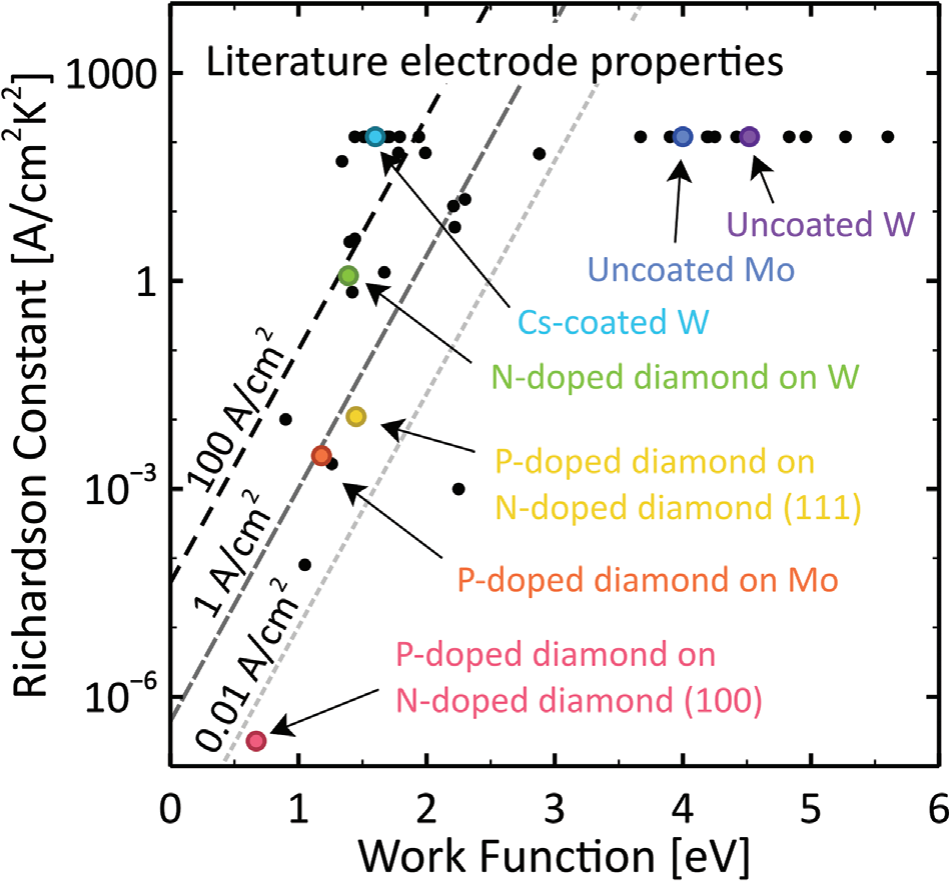
Work function and Richardson constant values for several TEC electrodes demonstrated in the literature.^[^ ^23^, ^40^, ^54^, ^65^, ^66^, ^67^, ^68^, ^69^, ^70^, ^71^, ^72^, ^73^ ^]^ Some values of interest are highlighted in color, and all data contained in this plot (black and colored dots) are available in Table 1. The dashed and dotted lines show the A‐value required to achieve a given emission current density as a function of the work function ϕ at $T_{e}=1500$ K according to Equation (2). Our choice of temperature reflects the fact that, at $T_{e}=1500$ K, work functions of $\phi_{e}=2.5$ eV and $\phi_{c}=1.5$ eV yield a core efficiency of η≈10% according to Figure 2. Note, however, that not all electrode materials represented in this figure can tolerate this temperature.

In addition, the electrode material properties must be chosen wisely. From Equation (2), in order to produce high current, the emitter must have a large Richardson constant and a relatively low work function $\left(\phi_{e}\;\left[\text{eV}\right]\approx T_{e}\;\left[K\right]/750\right)$; see **Figure** **4** and **Table** **1** for a selection of values from the literature,^[^ ^23^, ^40^, ^54^, ^65^, ^66^, ^67^, ^68^, ^69^, ^70^, ^71^, ^72^, ^73^ ^]^ as well as a compilation in Fomenko.^[^ ^74^ ^]^ It must simultaneously be stable at high temperatures ($T_{e}\approx$ 1000–2000 K) and ideally have a low thermal emissivity $\varepsilon_{e}$ to limit parasitic radiative heat transfer. The temperature and emissivity requirements on collector electrodes are not as stringent; however, they should have work functions substantially lower (by 0.5–1 eV) than their paired emitter, have surfaces that do not strongly reflect incoming electrons, and have low electrical resistivities to accommodate large currents through them.^[^ ^75^ ^]^ Unfortunately, few materials have been found that have work functions less than ≈1.5 eV in combination with low electron reflectivity and high‐temperature stability. To date, most TEC prototypes have achieved low work functions for their metallic electrodes using a submonolayer alkali metal (usually cesium) coating, which partially transfers electron charge from the adsorbate to the substrate and allows surface dipoles to form that lower the vacuum energy level near the surface.^[^ ^76^, ^77^, ^78^, ^79^ ^]^

**Table 1:** Selection of work function ϕ and Richardson constant A values for electrode materials available in the literature,^[^ ^23^, ^40^, ^54^, ^65^, ^66^, ^67^, ^68^, ^69^, ^70^, ^71^, ^72^, ^73^ ^]^ along with current density J achievable at $T_{e}=1500$ K according to Equation (2) (see also Fomenko^[^ ^74^ ^]^). Note, however, that not all electrode materials included in this table can tolerate this temperature. Abbreviations: NEA: negative electron affinity, C(D): diamond, C(PCD): polycrystalline diamond, C(NCD): nanocrystalline diamond

Substrate	Coating	Coating	NEA?	ϕ	A	J ($T_{e}=1500$ K)	Ref.
	layer 1	layer 2		[eV]	[Acm^−2^K^‐^^2^]	[Acm^−2^]	
Single‐crystal N‐doped C(D) with (100) surface orientation	P‐doped C(D)		yes	0.67	2.30$\times 10^{-7}$	2.90$\times 10^{-3}$	[23]
“Metallic substrate”	P‐doped C(PCD)		yes	0.9	1.0$\times 10^{-2}$	21.3	[68]
Quartz	W	BaO‐SrO‐CaO film	n/a	1.05	8.0$\times 10^{-5}$	5.34$\times 10^{-2}$	[54]
Mo	P‐doped C(PCD)		yes	1.18	3.0$\times 10^{-3}$	7.32$\times 10^{-1}$	[69]
Sapphire	W	BaO‐SrO‐CaO film	n/a	1.26	2.3$\times 10^{-3}$	3.02$\times 10^{-1}$	[54]
Mo with Re film	N‐doped C(NCD)	N‐doped C(D)	yes	1.34	53.1	3760	[71]
W	N‐doped C(NCD)	N‐doped C(D)	yes	1.39	1.19	57.2	[71]
Mo‐Re alloy	N‐doped C(NCD)	N‐doped C(D)	yes	1.4	3.67	163	[71]
Mo	N‐doped C(NCD)	N‐doped C(D)	yes	1.42	6.9$\times 10^{-1}$	26.3	[71]
Mo	N‐doped C(NCD)	N‐doped C(D)	yes	1.44	4.05	132	[69]
Nb	Cs		n/a	1.44	120	3920	[66]
Os	Cs		n/a	1.44	120	3920	[66]
Single‐crystal N‐doped C(D) with (111) surface orientation	P‐doped C(D)		yes	1.45	1.1$\times 10^{-2}$	3.32$\times 10^{-1}$	[23]
Re	Cs		n/a	1.51	120	2280	[66]
304 Stainless steel	Cs		n/a	1.52	120	2110	[65]
Pt	Cs		n/a	1.59	120	1230	[65]
W	Cs		n/a	1.6	120	1140	[66]
Mo	Cs		n/a	1.61	120	1050	[66]
Cu	Cs		n/a	1.64	120	834	[65]
“Metallic substrate”	N‐doped C(NCD)	N‐doped C(D)	yes	1.67	1.33	7.33	[72]
Ta	Cs		n/a	1.69	120	567	[66]
Cr	Cs		n/a	1.71	120	485	[65]
Ba‐activated W			n/a	1.78	70	165	[40]
Ir	Cs		n/a	1.79	120	261	[66]
Be	Cs		n/a	1.94	120	81.9	[65]
Si (*n*‐type)	N‐doped C(NCD)		yes	1.99	70	32.4	[67]
Single‐crystal N‐doped C(D) with (100) surface orientation			yes	2.21	12	1.01	[23]
Mo	N‐doped C(PCD)		yes	2.22	5.96	4.66$\times 10^{-1}$	[70]
Mo	N‐doped C(PCD)		no	2.25	9.97$\times 10^{-4}$	6.18$\times 10^{-5}$	[70]
Mo	P‐doped C(NCD)		yes	2.3	15	6.32$\times 10^{-1}$	[73]
Single‐crystal N‐doped C(D) with (100) surface orientation			yes	2.88	68	3.22$\times 10^{-2}$	[23]
Be			n/a	3.67	120	1.26$\times 10^{-4}$	[65]
Cr			n/a	3.9	120	2.13$\times 10^{-5}$	[65]
Mo			n/a	4	120	9.82$\times 10^{-6}$	[66]
Nb			n/a	4.19	120	2.26$\times 10^{-6}$	[66]
304 Stainless steel			n/a	4.2	120	2.09$\times 10^{-6}$	[65]
Ta			n/a	4.25	120	1.42$\times 10^{-6}$	[66]
Cu			n/a	4.42	120	3.81$\times 10^{-7}$	[65]
W			n/a	4.52	120	1.76$\times 10^{-7}$	[66]
Os			n/a	4.83	120	1.60$\times 10^{-8}$	[66]
Re			n/a	4.96	120	5.84$\times 10^{-9}$	[66]
Ir			n/a	5.27	120	5.31$\times 10^{-10}$	[66]
Pt			n/a	5.6	120	4.13$\times 10^{-11}$	[65]

### 2.4 Advanced Modeling

We note that the governing equations presented in Section 2.1 are sufficient to quantify the heat transfer modes introduced in Figure 1a for the case of an idealized micron‐gap TEC with negligible space charge (see the red and blue solid lines in the current–voltage characteristic plot of Figure 1b). To achieve output power density values of P>1 Wcm^−2^, electrode gaps should be reduced to roughly d<10 µm or other space charge mitigation strategies should be employed. Equations set forth in Hatsopoulos and Gyftopoulos^[^ ^13^, ^37^ ^]^ can be used to calculate the impact of space charge (see the black dashed lines in the current–voltage characteristic plot of Figure 1b). In addition, models often need to be augmented to include the impact of electron reflection from the collector^[^ ^75^ ^]^ and near‐field radiative heat losses.^[^ ^44^, ^63^, ^64^ ^]^ Other equations, such as those governing cesium plasmas or photovoltaic panels, are also necessary to describe variations from the essential TEC architecture introduced here. Still, the qualitative agreement between Figures 2 and 3 attests to the usefulness of the generalized model outlined in Section 2.1. In addition, numerous publications with model‐experiment comparisons provide further evidence of its applicability.^[^ ^40^, ^46^, ^52^, ^56^ ^]^

## 3 Historical Research

### 3.1 Plasma‐Based Converters

Significant research effort in the mid‐twentieth century worked to address the space charge problem using plasmas. As mentioned above, positively‐charged plasmas introduced to the intragap space partially neutralize the electron‐induced electric field, allowing higher currents to be achieved. Plasma‐based devices, while successful in delivering reasonable power densities (P≈ 10–20 Wcm^−2^) for long time intervals (>50 000 h), were largely limited to moderate efficiencies (η≈ 6–13%), in part due to the energy required to sustain the plasma (this was referred to as the *ignited mode* of operation).^[^ ^11^, ^12^, ^18^ ^]^

**Figure 5:**
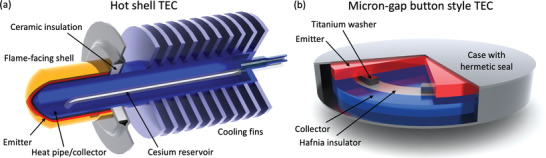
a) Schematic diagram of a hot shell thermionic energy converter (based on a figure by Veltkamp et al.; rendition included with permission.^[^ ^82^ ^]^ Original figure copyright 1989, IEEE). Hot combustion gases heat the outer shell, which conducts heat to the emitter. The central heat pipe, the outer surface of which also serves as the collector, transfers heat away from the shell to outside air with the assistance of external cooling fins. b) Schematic diagram of a twentieth century micron‐gap button‐style thermionic energy converter (based on a figure by Beggs^[^ ^51^ ^]^). A titanium washer and ceramic insulator are used to separate the emitter and collector, and the entire device is hermetically sealed. Combustion gases heat the emitter side of the device, and the collector side dissipates heat to the air through natural convection.

A particularly noteworthy design was a plasma‐based TEC developed by Anderson and Horner–Richardson that achieved an estimated efficiency of η=16% at $T_{e}=2200$ K (no power density was provided).^[^ ^80^ ^]^ Another example was a plasma‐based converter reported by Smith, Manda, and Britt that achieved an output power density of P=6 Wcm^−2^ at $T_{e}=1600$ K (no efficiency was given).^[^ ^81^ ^]^ Also important were combustion‐heated hot shell emitter style TECs (**Figure** **5**a), in which hot gases impinged upon the top of a dome‐shaped protective silicon carbide layer and heat subsequently transferred into a tungsten emitter on the inside of the dome. The devices were able to achieve current and output power densities of A≈5−10 Acm^−2^ and P≈2.5−5 Wcm^−2^, respectively.^[^ ^3^, ^82^, ^83^, ^84^ ^]^

Experimentation with plasma‐based TECs was largely discontinued after the twentieth century, due in part to the complexity of plasma engineering and the efficiency limitations of these devices that required them to be operated at very high temperatures. We refer the reader to the previous literature reviews for additional information.^[^ ^9^, ^12^, ^14^, ^15^, ^61^, ^62^ ^]^

### 3.2 Twentieth Century Micron‐Gap Converters

Several engineers sought simpler designs to address the space charge problem while avoiding the need for plasma. Perhaps the most important product of this work were micron‐gap converters, whose micrometer‐scale electrode spacings reduced the number of electrons in transit, thereby minimizing their mutual repulsion through the resulting electric field. An early example was a d=25 µm gap TEC developed by Hatsopoulos and Kaye that produced an output power density of P=0.76 Wcm^−2^ at an estimated efficiency of 13% when operating at an emitter temperature of $T_{e}=1538$ K.^[^ ^49^, ^50^ ^]^ The authors noted the “arduous efforts” and “meticulous handling” required to complete these experiments, which involved moving the delicate electrodes on sliders within a vacuum jar.^[^ ^50^ ^]^ In contrast to these chamber‐based experiments, Beggs designed several fully encapsulated button‐style TECs that featured washer‐style ceramic and metal gap‐maintaining spacers (Figure 5b). These could be handled easily and exposed directly to flames for heating. A prototype with a gap of d=6 µm produced an output power density of about P=1.1 Wcm^−2^ at an estimated efficiency of η=4.5% when heated to $T_{e}=1373$ K. The difference in efficiency values between the chamber‐based prototype of Hatsopoulos and Kaye^[^ ^49^, ^50^ ^]^ and the encapsulated TECs of Beggs^[^ ^51^ ^]^ likely stems from the excessive conduction present in the latter designs, highlighting the importance of accounting for parasitic conduction in modeling and design efforts. Importantly, however, Beggs noted that power output increased as the gap distance decreased, consistent with a reduction in space charge effects.^[^ ^51^ ^]^

Additionally, Dick, Britt, and Fitzpatrick reported several TECs (called SAVTEC for *self‐adjusting versatile thermionic energy converter*) whose microgaps, in the range d=6−13 µm, were created by thermal expansion. The emitters of these devices were heated using combustion to temperatures between 1100 and 1750 K, resulting in estimated efficiencies between 3.3% and 18%.^[^ ^52^, ^53^ ^]^ One prototype operating at $T_{e}=1800$ K produced a power density of P=4.2 Wcm^−2^ while operating in the Knudsen mode,^[^ ^85^ ^]^ in which cesium ions ejected from the cathode neutralized space charge effects (note that this is different from plasma ignited mode operation).^[^ ^52^ ^]^ Another variant with a gap of approximately d=11.9 µm operating at a lower temperature of $T_{e}=1453$ K (not in the Knudsen mode) produced a power density of P=0.341 Wcm^−2^.^[^ ^52^ ^]^ Unfortunately, the authors noted that in some cases the TECs experienced electrical shorts due to inadequate thermal expansion to separate the emitter and collector.^[^ ^52^, ^53^ ^]^ In a subsequent study, Fitzpatrick, Nikolaev, and McVey experimented with a different TEC whose microgap of d=10 µm was maintained by alumina ceramic spacers. When operated at $T_{e}=1300$ K, it produced a power density of P=0.370 Wcm^−2^ at an estimated efficiency of η=11.6%.^[^ ^46^ ^]^

The primary difficulties associated with micron‐gap TECs were in maintaining consistently small interelectrode distances despite thermal expansion and in limiting parasitic thermal conduction through spacers or external supports. Importantly, however, these prototypes demonstrated the feasibility of overcoming space charge effects without using plasma.

## 4 Novel Electrode Materials and Designs

Many new ideas for improving the performance of thermionic energy converters have been proposed recently. Here we present and critique novel electrode materials and designs in terms of their ability to produce power densities of P≈1 Wcm^−2^, a threshold value for practical device operation.

### 4.1 Carbon Nanotube Emitters

One proposed route to increase the electrical conversion efficiency of TECs is to reduce the conductive heat transfer losses to structural device elements using carbon nanotube (CNT) forests as emitters.^[^ ^86^, ^87^, ^88^, ^89^, ^90^, ^91^, ^92^ ^]^ In this approach, a spot of solar or laser light focused on the side of the CNT forest produces local heating, facilitated by the fact the tubes' thermal conductivity decreases as their temperature increases. In one case, sunlight was focused onto the side of a CNT forest to a 700‐µm diameter spot, raising the mean temperature to $T_{e}>2000$ K. Unfortunately, the CNT emitter prototypes demonstrated thus far have achieved efficiencies of only a fraction of a percent due to their high work functions (4 to 5 eV) and space charge‐related issues. For instance, assuming a CNT forest with a Richardson constant of $A_{e}=120$ Acm^−2^K^‐^^2^, a work function of $\phi_{e}=4.6$ eV, and a temperature of $T_{e}=2000$ K, Equation (2) predicts a current density of only about J≈1 mAcm^−2^. Though efforts to reduce CNT work functions through the intercalation of potassium have had some success,^[^ ^93^, ^94^ ^]^ additional research is needed to demonstrate that carbon nanotube emitters can produce current densities of practical importance (≈1 Acm^−2^).

### 4.2 Textured Electrodes

Aside from carbon nanotube emitter‐based TECs, most prototypes have employed smooth electrode surfaces obtained by using precision grinding or micromachining techniques. However, some researchers have experimented with emitters that have engineered surface textures such as bump arrays or waviness.^[^ ^95^, ^96^, ^97^ ^]^ These features can raise the emitted electron current density (plane‐averaged) by both increasing the total emitter surface area and through Schottky barrier lowering due to intensified local electric fields near the protrusions. Collector electrodes can also be textured; such geometry can serve to trap electrons and reduce the reverse (collector to emitter) electron flow.^[^ ^98^ ^]^ Notably, recent work by Fernandes Cauduro et al. has shown that nanoscale order can be exploited to create an “electron‐black” electrode surface that suppresses reflection. Using standard thermionic materials, this nanoengineering reduced overall reflection from >20% for a crystalline surface to roughly 4% with texture.^[^ ^99^ ^]^

A majority of the experimental research has explored textured electrodes in cesiated TECs with macro‐scale grooves (≈500 µm depth) and large electrode gaps (d≈1 mm).^[^ ^100^, ^101^, ^102^, ^103^, ^104^ ^]^ We are aware of one experimental study examining textured electrodes in non‐cesiated micron‐gap configurations,^[^ ^105^ ^]^ though three modeling studies have been published.^[^ ^75^, ^106^, ^107^ ^]^ Electrodes with nanoscale texture have also been fabricated using diamond films^[^ ^106^, ^108^ ^]^ and tungsten plates.^[^ ^109^ ^]^ Additional research is needed to optimize manufacturing, material, and texture‐geometry‐related aspects of this technology to achieve practical output power densities.

### 4.3 Plasmonic Thermionic Converters

**Figure 6:**
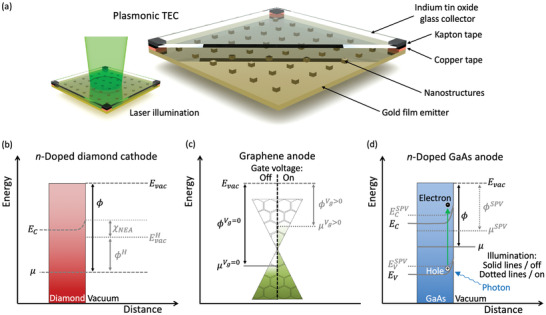
a) Schematic diagram of a plasmonic thermionic energy converter. The surface of the emitter contains nanoscale protrusions that, when exposed to light (see inset), promote a subpopulation of excited electrons with a characteristic temperature roughly one order of magnitude higher than that of the bulk material, thereby enhancing the thermionic emission toward the transparent collector plate above. Figure based on a diagram by Wu, Hogan, and Sheldon.^[^ ^111^ ^]^ b) Electron motive diagram for a *n*‐doped diamond cathode with Fermi level μ and conduction band energy minimum $E_{C}$, without (black text) and with (gray text) hydrogen surface passivation. Upon hydrogen passivation, the C‐H terminations establish a negative electron affinity $\chi_{\text{NEA}}$ and the vacuum level is reduced to $E_{\text{vac}}^{H}$, effectively lowering the work function to $\phi^{H}$. Figure based on a graphic by Takeuchi et al.^[^ ^112^ ^]^ c) Dirac cone diagram showing how electrostatic gating modifies a graphene electrode's work function. When the gate voltage is applied ($V_{g}>0$ V), compensating charges accumulate in the graphene and the Fermi level μ increases relative to the vacuum level $E_{\text{vac}}$, thereby decreasing the work function ϕ.^[^ ^58^, ^113^ ^]^ Note that this technique can be used in combination with an alkali metal coating on the electrode surface to reduce the vacuum energy level (not shown in this figure). d) Electron motive diagram showing the surface photovoltage effect (SPV) for a *n*‐doped GaAs anode with vacuum energy level $E_{\text{vac}}$. When the sample is illuminated, the incident photons create electron–hole pairs, reducing the band bending (represented by the position‐dependent valence band maximum and conduction band minimum energy levels $E_{V}^{\text{SPV}}$ and $E_{C}^{\text{SPV}}$, respectively), increasing the Fermi level to $\mu^{\text{SPV}}$, and reducing the work function to $\phi^{\text{SPV}}$. Figure based on a graphic by Schindler et al.^[^ ^79^ ^]^

Surface texture can also be used to alter electronic temperatures and thereby enhance emission, as demonstrated recently by Wu, Hogan, and Sheldon in a device called a plasmonic thermionic converter (**Figure** **6**a).^[^ ^110^, ^111^ ^]^ In these devices, concentrated light is directed onto a metallic emitter whose surface contains nanoscale raised protrusions. This texture increases the local light absorption, which causes surface plasmons, or coherent oscillations of free electrons, to form. Subsequent electron–electron scattering creates a subpopulation of excited electrons whose temperature can be more than an order of magnitude greater than that of the associated metal lattice. According to the Richardson–Dushmann equation, some of these excited electrons are free to escape the emitter surface and cross a vacuum gap to a collector as in a traditional TEC. The authors note that an advantage of this approach is that it allows emitters at lower bulk material temperatures to generate significant emission through higher effective electron temperatures, thereby alleviating material constraints and decreasing parasitic conduction and radiation heat transfer losses. Though the proof‐of‐concept prototype was successful, its maximum power density was only P=2 µWcm^−2^ and its core efficiency was $\eta \approx 1.2\times 10^{-7}$%, as expected from the prototype's large 200‐µm gap distance and high $\phi_{e}=5.1$ eV emitter work function. Future plasmonic TECs should address several engineering challenges in order to be commercially viable, such as using electrodes with lower work functions, developing efficient optically transparent collectors (anodes), and identifying emitter nanostructures that allow the entire solar spectrum, rather than a single laser wavelength, to be harvested.

### 4.4 Diamond Electrodes

According to Equation (2), an important route toward increasing electron emission and thereby boosting TEC performance is to reduce the electrode work functions ($\phi_{e}$ and $\phi_{c}$). As shown in the insets shown in Figure 1b, this can be accomplished by either reducing electrodes' vacuum energy level $E_{\text{vac}}$ or increasing their Fermi level μ:

(14) $$\phi =E_{\text{vac}}-\mu$$

The vacuum energy level of an electrode is typically reduced by surface engineering, a classic example of which is applying a cesium coating to a refractory metal (e.g., tungsten) cathode, which creates a surface dipole layer.^[^ ^14^, ^76^, ^114^ ^]^ Surface engineering can also be accomplished using diamond electrodes with hydrogen passivation layers.^[^ ^23^, ^68^, ^70^, ^71^, ^72^, ^73^, ^112^, ^115^, ^116^, ^117^ ^]^ Electrodes composed of diamond, when properly doped with nitrogen or phosphorus (i.e., *n*‐type doping) and subsequently passivated using hydrogen to induce a negative electron affinity (NEA), have demonstrated work functions as low as ϕ=0.67 eV.^[^ ^23^ ^]^ The negative electron affinity means that the vacuum energy level is lower than the conduction band minimum (CBM) energy, such that valence band electrons can be excited directly to the vacuum without having to overcome a surface emission energy barrier (Figure 6b).^[^ ^112^ ^]^ Some modeling studies have also argued that a negative electron affinity collector could mitigate or eliminate space charge effects under some conditions.^[^ ^118^, ^119^, ^120^ ^]^

Unfortunately, while diamond itself is stable at high temperatures, the hydrogen passivation layer has been found to deteriorate at temperatures in the range $800<T_{e}<1100$ K.^[^ ^70^, ^121^ ^]^ This was experimentally investigated by Paxton et al.,^[^ ^70^ ^]^ who heated a hydrogen‐passivated diamond film grown on a molybdenum substrate and measured the emitted current. The current initially increased according to the Richardson–Dushmann equation (Equation (2)) to a peak of approximately 23 nA (no emission area was provided), but then sharply decreased at temperatures in excess of about 1075 K, corresponding to deterioration of the hydrogen passivation layer. Additionally, diamond and other materials with remarkably low work functions also exhibit extremely low Richardson constants; for instance, the ϕ=0.67 eV emitter mentioned above had $A=2.3\times 10^{-7}$ Acm^−2^K^‐^^2^,^[^ ^23^ ^]^ which is many orders of magnitude lower than those of commonly used cesiated (i.e., cesium‐coated) metals (see Figure 4 and Table 1). Though such electrodes may be intended to function as electron collectors rather than electron emitters, low A‐values are associated with elevated resistivity values and may lead to excessive reflection of incident electrons.^[^ ^23^, ^25^, ^71^, ^75^ ^]^ In order for hydrogen‐passivated diamond electrodes to be practical for commercial electricity generation, research must be devoted to maintaining stable operation at temperatures of 1300–1500 K (for use as emitters) and toward achieving higher electrical conductivity and low electron reflection (for use as collectors).

### 4.5 Suspended Graphene Cathodes

In common 3D materials, the Richardson‐Dushmann equation (Equation (2)) has a $T^{2}$ pre‐factor, which has been found accurate for numerous metallic substrates. However, recent analytical derivations have asserted that graphene sheets and other select materials such as 3D Dirac semimetals may exhibit a pre‐factor temperature dependence of $J\approx \;T^{3}$.^[^ ^122^, ^123^, ^124^, ^125^, ^126^, ^127^, ^128^, ^129^ ^]^ Several researchers have proposed that this enhanced temperature dependence could prove advantageous if such materials were used as emitters in TECs.^[^ ^122^, ^126^, ^128^, ^129^ ^]^ However, we are aware of only a single dataset^[^ ^130^ ^]^ that has been used to experimentally validate this enhanced temperature dependence,^[^ ^122^, ^124^ ^]^ and we are unaware of any TEC prototypes that have been created to demonstrate this concept. In addition, the derivations of this enhanced emission^[^ ^122^, ^124^, ^129^ ^]^ typically consider the density of electron states in the material alone, rather than considering the maximum current density possible in the vacuum; this can in some cases yield higher‐than‐feasible emission current densities.^[^ ^13^, ^39^ ^]^ Additional experimental investigations and proof‐of‐concept prototypes are needed to evaluate the feasibility of this approach.

### 4.6 Graphene Anodes

According to Equation (14), the work functions of electrodes can be reduced by either lowering their associated vacuum energy level or by increasing their Fermi level. The combination of these two approaches was experimentally demonstrated recently by Yuan et al., who voltage‐biased a graphene collector (anode) such that it accumulated compensating charges in a process called electrostatic gating (Figure 6c).^[^ ^58^, ^113^, ^131^ ^]^ The researchers employed this technique, which increased graphene's Fermi level, together with a Cs/O coating on the graphene, which reduced its vacuum energy level, to lower graphene's work function to ϕ=1.01 eV.^[^ ^58^ ^]^ The authors subsequently constructed a micron‐gap (d=17 µm) TEC using a gated and cesiated graphene anode collector with $\phi_{c}=1.69$ eV that produced a power density of about P=13 mWcm^−2^ with a core efficiency of about η=0.09% at $T_{e}=1273$ K. These low power density and efficiency values reflect the large gap distance and high parasitic heat transfer values experienced by the prototype, which could be addressed in future designs.^[^ ^113^ ^]^

### 4.7 Surface Photovoltage Effect

Another method to lower work functions involves the surface photovoltage effect. In a recent study, a *n*‐type GaAs semiconductor electrode with a Cs/O coating was illuminated with 532‐nm laser light (Figure 6d).^[^ ^79^ ^]^ The incident photons generated electron‐hole pairs that reduced the band bending near the semiconductor surface (surface photovoltage, SPV) and increased the Fermi level such that the work function was lowered by between 0.2 and 0.4 eV (see Equation (14)). The authors constructed a proof‐of‐concept TEC using this electrode as a collector (anode) but using a Ba coating rather than Cs/O and 650‐nm laser illumination rather than 532 nm. The experiments achieved an open‐circuit current of roughly J=33 µAcm^−2^ with an emitter temperature of $T_{e}=888$ K and a collector work function of $\phi_{c}=1.8$ eV. We note that, to be practical for at‐scale electricity production, anodes based on the surface photovoltage effect must be capable of sustaining much higher current density values.

### 4.8 Photon‐Enhanced Thermionic Emission

**Figure 7:**
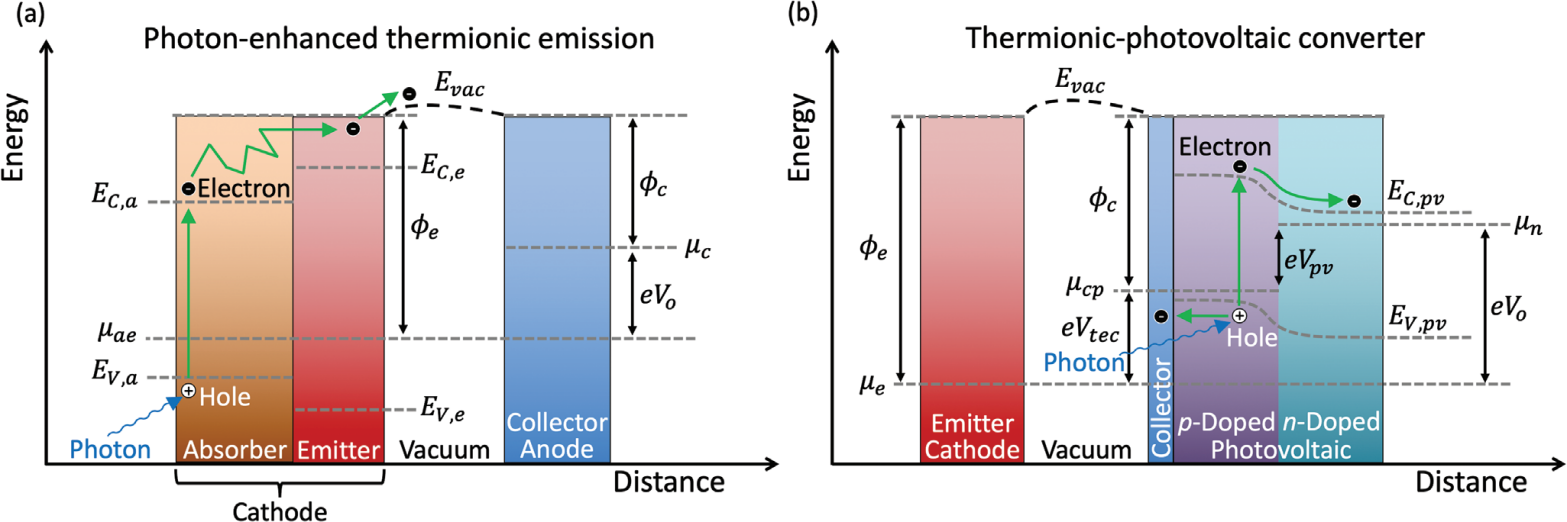
a) Electron motive diagram showing photon‐enhanced thermionic emission (PETE) with a heterostructure emitter. Photons incident upon the absorber excite valence electrons with energy $E<E_{\text{V,a}}$ to the absorber's conduction band ($E>E_{\text{C,a}}$) where they thermalize; sufficiently energetic electrons subsequently diffuse to the emitter's conduction band ($E>E_{\text{C,e}}$), from which a fraction with $E>E_{\text{vac}}$ can enter the vacuum gap and travel to the collector. In this illustration, $\phi_{e}$ and $\phi_{c}$ are the work functions of the emitter and collector, respectively, $\mu_{\text{ae}}$ and $\mu_{c}$ are the Fermi levels of the absorber‐emitter and collector, respectively, $eV_{o}$ is the output voltage scaled by the elementary charge, and $E_{\text{V,e}}$ is the valence level maximum energy of the emitter. Figure based on a graphic from Schwede et al.^[^ ^132^, ^133^ ^]^ b) Electron motive diagram of a hybrid thermionic‐photovoltaic (TIPV) converter. Electrons and photons, both produced in the emitter, simultaneously transit the gap and enter the collector and photovoltaic cell, respectively. The incident electrons combine with holes produced by the incident photons. The electrons that formerly occupied those holes are promoted to the conduction band of the photovoltaic cell (with position‐dependent minimum energy $E_{\text{C,pv}}$) and diffuse to the back of the *n*‐doped side of the photovoltaic cell. In this illustration, $\phi_{e}$ and $\phi_{c}$ are the work functions of the emitter and collector, respectively; $\mu_{e}$, $\mu_{\text{cp}}$, and $\mu_{n}$ are the Fermi levels of the emitter, collector‐*p*‐doped side of the photovoltaic cell, and *n*‐doped side of the photovoltaic cell, respectively; $eV_{\text{tec}}$, $eV_{\text{pv}}$, and $eV_{o}$ are elementary charge‐scaled output voltages of the thermionic section, photovoltaic cell, and total converter, respectively; $E_{\text{vac}}$ is the position‐dependent vacuum energy level; and $E_{\text{V,pv}}$ is the position‐dependent valence level maximum energy of the photovoltaic cell. Figure based on a graphic by Datas et al.^[^ ^59^, ^148^, ^149^ ^]^

A different method for effectively shifting Fermi levels that is possible in solar applications is photon‐enhanced thermionic emission (PETE), which combines the thermionic and photovoltaic effects by using the per‐quanta energy of photons to alter the electron energy distribution within the emitter and drive the electron flow (**Figure** **7**a). This combination theoretically improves upon traditional photovoltaic devices by thermally using the energy of sub‐bandgap photons and of photons with energy in excess of the semiconductor bandgap, and improves upon pure TEC designs by enhancing the thermionic emission flux beyond that expected by the Richardson‐Dushmann equation (Equation (2)). The concept was experimentally demonstrated by Schwede et al.^[^ ^132^, ^133^ ^]^ and was reviewed recently by Kribus and Segev^[^ ^134^ ^]^; early related work was performed by Smestad.^[^ ^135^ ^]^ While numerous experiments have explored emitter materials for PETE,^[^ ^136^, ^137^, ^138^, ^139^ ^]^ and several modeling studies have proposed optimal PETE configurations,^[^ ^140^, ^141^, ^142^, ^143^, ^144^, ^145^, ^146^, ^147^ ^]^ none have demonstrated complete energy conversion systems that can operate at elevated temperatures.^[^ ^134^ ^]^ For instance, the heterostructure emitter quantum efficiency measurements conducted by Schwede et al. were limited to roughly $T_{e}\approx 400$ K.^[^ ^133^ ^]^ Primary difficulties include overcoming electron‐hole recombination in the emitter and engineering coatings and photovoltaic materials that can withstand high emitter temperatures.

### 4.9 Hybrid Thermionic–Photovoltaic Converters

Related to the two previous examples of photon‐based enhancements of converter performance, recently, Datas et al. proposed a combined thermionic and thermophotovoltaic converter^[^ ^59^, ^150^ ^]^ (see also two earlier related proposals^[^ ^151^, ^152^ ^]^). Recall that thermophotovoltaic converters (TPVs) transform thermal radiation from an emitter into electricity using a photovoltaic receiver.^[^ ^33^ ^]^ Improving on this concept, Datas et al.'s hybrid thermionic–photovoltaic (TIPV) device, shown in Figure 7b, features a cathode that emits both photons and electrons, a vacuum micron‐scale gap, and a photovoltaic receiver with a transparent electron‐collecting thin‐film coating.^[^ ^59^, ^150^ ^]^ Two potential advantages of this design are that it allows the radiative emission from the emitter to contribute to the output power and that the electrons collected on the anode combine with holes in the photovoltaic cell, eliminating the need for lateral current flow through collecting grids.^[^ ^148^, ^149^, ^150^, ^153^ ^]^ A prototype constructed by Bellucci et al. operated between electrodes at approximately $T_{e}=1700$ K and $T_{c}=300$ K and yielded a power density of only ≈1 µWcm^−2^, a low value reflecting the large gap distance (d=125 µm) and high electrode work functions ($\phi_{e}\approx 4.6$ eV and $\phi_{c}\approx 4$ eV).^[^ ^59^ ^]^

A number of improvements must be made to make TIPV technology feasible for practical energy generation. These include achieving a better match between the absorption threshold of the photovoltaic cell and the infrared emission of the emitter, reducing the photon absorption losses in the collector layer, further reducing the electrode work functions, and developing a photovoltaic cell that can withstand elevated temperatures in order to eliminate excessive collector cooling requirements. Simulations suggest that this architecture can produce high power density in a wider temperature range than single thermionic or thermophotovoltaic converters alone, indicating its utility in applications where the input temperature is variable.^[^ ^154^, ^155^, ^156^, ^157^ ^]^ Additionally, recent calculations have addressed the issue of cooling the anode to avoid overheating the photovoltaic cell.^[^ ^158^ ^]^

### 4.10 Summary of Electrode Research

To summarize, research work related to electrode materials and designs can be grouped into schemes to mitigate heat transfer (carbon nanotube emitters), methods to manipulate the electrode surface geometry or coating (textured electrodes, plasmonic TECs, and diamond electrodes), strategies to employ 2D materials (graphene cathodes and anodes), and hybrid architectures that rely on both electrons and photons (surface photovoltage effect, photon‐enhanced thermionic emission, and hybrid thermionic–photovoltaic converters). As emphasized, continued effort is needed to achieve high power density values using many of these approaches. We will recommend a few strategies for further research in Section 6.

## 5 New Methods to Mitigate Space Charge

While robust electrodes with low work functions serve an important role in thermionic energy conversion, another significant aspect of enabling high power density involves mitigating space charge effects, which reduce the current density relative to the ideal case (Figure 1b). In this section we outline several efforts to address space charge that differ from the traditional plasma‐based charge neutralization strategy.^[^ ^9^, ^12^, ^14^, ^15^, ^61^, ^62^ ^]^

### 5.1 Solid‐State Converters

Solid‐state thermionic devices are converters in which the vacuum gap has been replaced with a solid material barrier, most frequently a semiconductor. (The name is somewhat confusing in that, for the case of generic TECs, the adjective *solid‐state* refers to their lack of moving parts, whereas the term *solid‐state TECs* is used in reference to TECs that have no vacuum gap.) The concept was introduced in the context of compression‐cycle‐free refrigeration, but has been discussed in applications of waste heat recovery as well.^[^ ^159^, ^160^, ^161^, ^162^, ^163^, ^164^ ^]^ Solid‐state TECs avoid space charge limitations because they frequently employ very thin barriers (width d≈1−100 nm) and also dope the semiconductor barrier to limit its band bending. Another advantage relative to vacuum gap TECs is that the barrier height for electron emission from the cathode can be decreased by tuning the semiconductor barrier's electron affinity, such that practical emission currents can be obtained at lower temperatures. Unfortunately, solid‐state TECs' lack of a vacuum gap results in large parasitic conductive losses as compared to the relatively small conductive and radiative losses in vacuum micron‐gap converters (see Figure 2).^[^ ^165^ ^]^ Solid‐state TECs can be regarded as being similar to thermoelectric converters, which rely on the Seebeck effect, the significant difference being that electrons transit through the semiconductor ballistically in solid‐state TECs whereas they move diffusively in thermoelectric devices. A succinct but helpful discussion is provided by Vining and Mahan,^[^ ^164^ ^]^ who found solid‐state thermionic converters to be less efficient than thermoelectric converters in the limit of small temperature differences. Recent research efforts have focused on developing new semiconductor materials that limit conductive heat transfer, including van der Waals heterostructures of transition metal dichalcogenides.^[^ ^166^, ^167^, ^168^, ^169^ ^]^ Predicted efficiency values for solid‐state TECs recovering waste heat are often of order η≈1−10%, though few, if any, experimental studies have approached these targets.^[^ ^168^ ^]^ We refer the reader to two recent reviews for more information.^[^ ^170^, ^171^ ^]^

### 5.2 Improved Plasma‐Based Converters

As discussed above, many TEC prototypes of the twentieth century used an intragap plasma to produce cesium ions to neutralize the space charge, with a significant drawback being the efficiency penalty associated with maintaining the energetic discharge. To circumvent this, several articles have proposed alternative methods to generate cesium ions or configurations to improve the performance of plasma‐based converters. Hatsopoulos and Gyftopoulos^[^ ^13^ ^]^ reviewed several of these, including cesium diodes with electronegative and electropositive additives, pulsed diodes, radiation diodes, ion emission vapor triodes, and arc triodes. Efforts involving electronegative additives (e.g., oxygen) and electropositive additives (e.g., barium) included those of Psarouthakis^[^ ^172^ ^]^ and Desplat, Rasor, and Dobson.^[^ ^173^ ^]^ In addition, Rasor proposed several designs, including a differentially‐heated cesium‐oxygen reservoir^[^ ^174^ ^]^ and multi‐material emitters that used high work function regions to ignite a plasma that mitigated space charge from electrons emitted from other low work function regions.^[^ ^175^, ^176^ ^]^ Moyzhes and Geballe devised and simulated a TEC in which a third electrode with a high work function, situated between the emitter and collector, generated cesium ions.^[^ ^177^ ^]^ Also, a study by Mustafaev et al. examined the impact of feeding cesium vapor through a porous nickel collector.^[^ ^178^ ^]^ Finally, Zheng, Ogino, and Kando constructed a TEC in which a xenon lamp (a substitute for sunlight) was used to ionize cesium, generating a current density of J≈30 mAcm^−2^ at an emitter temperature of $T_{e}\approx 1500$ K.^[^ ^179^ ^]^ Further development is necessary to test these concepts under practical operating conditions to achieve high output power densities and reasonable efficiencies.

### 5.3 Gate‐Assisted Converters

**Figure 8:**
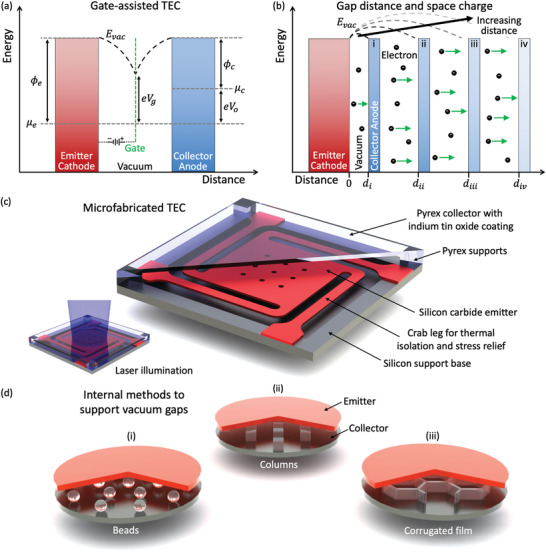
a) Electron motive diagram of a gate‐assisted thermionic energy converter. An electron‐transparent gate with an elementary charge‐scaled voltage bias of $eV_{g}$ is placed in between the emitter and collector in order to reduce the space charge, and a fraction of the electrical power output is directed to maintain its potential. In this figure, $\phi_{e}$ and $\phi_{c}$ are the work functions of the emitter and collector, respectively; $\mu_{e}$ and $\mu_{c}$ are the Fermi levels of the emitter and collector, respectively; and $E_{\text{vac}}$ is the position‐dependent vacuum energy level. Figure based on a graphic by Wanke et al.^[^ ^180^ ^]^ (included with permission from AIP Publishing). b) Illustration of the relationship between gap distance and space charge, for a TEC operating at the maximum power point. As the collector distance is increased from $d_{i}$ to $d_{\text{iv}}$, the maximum energy of the position‐dependent vacuum level $E_{\text{vac}}$ increases; this increases the effective energy barrier and reduces the electron current that reaches the collector. c) Schematic diagram of a microfabricated thermionic energy converter (based on a figure by Lee et al.^[^ ^188^ ^]^). The silicon carbide emitter is suspended over a lower silicon support base to limit conductive heat transfer losses and allow for thermal expansion. The collector, composed of optically transparent Pyrex with a conductive indium tin oxide coating, is fabricated separately and bonded above the emitter. The inset shows how the emitter is optically heated (in this case using 455‐nm laser light) through the transparent collector. Note that a prior version of this device featured a silicon collector below the emitter.^[^ ^189^ ^]^ d) Schematic diagrams illustrating three methods of maintaining micron‐scale vacuum gaps: i) beads (similar to Littau et al.^[^ ^40^ ^]^), ii) columns (similar to Belbachir, An, and Ono^[^ ^57^ ^]^ and Bellucci et al.^[^ ^60^ ^]^), and iii) freestanding corrugated films (similar to Nicaise et al.^[^ ^190^ ^]^).

The two primary strategies mentioned thus far for addressing the space charge problem have involved plasmas and micron‐sized gaps. A third strategy, similar to a vacuum triode, uses an element called a *gate* or *grid* that is positioned between the electrodes to modify the electric field and accelerate electrons toward the collector^[^ ^180^, ^181^, ^182^ ^]^ (**Figure** **8**a; see also a related patent^[^ ^183^ ^]^); we will refer to this as a gate‐assisted thermionic energy converter. In addition, a magnetic field, directed along the direction of the electron trajectories, reduces the frequency of electrons encountering this middle electrode. An advantage of this architecture is that larger emitter‐collector gap distances can be used, easing manufacturing constraints and allowing better conductive insulation; however, the power required to sustain the gate's electric field directly reduces the device's total output current density.

This concept was experimentally demonstrated in a device with a tungsten foil gate and BaO dispenser cathodes for both the emitter and collector by Meir et al.^[^ ^56^ ^]^; the gate‐assisted TEC produced a power density of P=13 mWcm^−2^ at a current density of J=14 mAcm^−2^ with a gap distance of d≈500 µm, electrode temperatures of $T_{e}=1373$ K and $T_{c}=773$ K, and a gate voltage of $V_{g}=+6$ V. Modifications of this concept using electron‐transparent graphene gates have been proposed^[^ ^180^, ^184^ ^]^ and a variant using two gates has been tested.^[^ ^185^ ^]^ The gate‐assisted converter geometry has also been used to measure surface work functions,^[^ ^186^ ^]^ and others have modeled its use in solar concentrating applications.^[^ ^187^ ^]^ Additional materials science and engineering difficulties for the gate‐assisted converter strategy remain to be solved, most significantly developing gates with greater electron transparencies; however, the existence of a functional prototype that achieved at least mAcm^−2^ current densities^[^ ^56^ ^]^ is promising for future developmental efforts.

### 5.4 Modern Micron‐Gap Converters

One important development of the 1990s and 2000s was the ability to create electrodes and micron‐sized gaps using microfabrication techniques, which are more precise than precision machining methods. As mentioned above, microgaps minimize the number of electrons in transit between the emitter and collector, thus mitigating the space charge effect (Figure 8b). A prototype fabricated by King, Luke, and Zavadil with a gap distance of d≈20 µm produced a power density of P=890 µWcm^−2^ at $T_{e}=1170$ K,^[^ ^54^ ^]^ and a similar prototype developed by Zhang et al. with a gap distance of d≈10 µm and operated at the same emitter temperature yielded a power density of P=900 nWcm^−2^ (the authors of the latter paper observed that electrical connections in their device could have been faulty).^[^ ^55^ ^]^ In addition, a microfabricated TEC developed by Lee et al. featured a silicon carbide emitter with a silicon collector separated by a gap distance of d≈10 µm; it produced a power density of roughly P=3.6 µWcm^−2^ at a core efficiency of approximately $\eta =3\times 10^{-6}$%.^[^ ^189^ ^]^ In a later study, Lee et al. modified their design to incorporate a barium emitter coating and an indium tin oxide‐coated Pyrex collector, achieving an estimated efficiency of η=0.5% (Figure 8c).^[^ ^188^ ^]^ Other TECs with gap distances of 1.7<d<100 µm have been fabricated as well.^[^ ^191^, ^192^ ^]^ Unfortunately, many of these microelectromechanical systems (MEMS)‐inspired designs were hampered by thermal stresses, excessive parasitic heat conduction, or problems with electrode coatings that produced suboptimally large work functions.

The previous microfabricated prototypes established thin gaps using external structures. Small gaps can also be achieved using intragap structures such as insulating spacers that are in contact with the emitter and collector. Spacers allow the tight tolerances of externally‐supported TEC stacks, such as those of the previous paragraph, to be relaxed, because they impose a well‐defined electrode spacing internally. Importantly, spacers must be electrically and thermally insulating, while simultaneously allowing a high geometrical area for electron transmission.

Littau et al. placed alumina ceramic beads between a barium‐impregnated tungsten emitter and a tungsten‐film‐coated silicon wafer collector (Figure 8d‐i).^[^ ^40^ ^]^ With a gap of roughly d≈11 µm, the device produced a maximum power density of P=290 mWcm^−2^ at an estimated efficiency of 0.61%. These low values reflect radiation heat transfer losses through the sides of the emitter, conductive losses through the leads, and the relatively high collector work function ($\phi_{c}=1.8$ eV). In addition, the researchers noted that the bead sizes had a relatively wide distribution, resulting in uncertainty about the exact gap distance.

Another example of an internally‐supported interelectrode gap is that of Belbachir, An, and Ono, who used micro‐fabricated silicon dioxide columns to separate a silicon carbide emitter from a thin platinum film collector by a distance of d=10 µm (Figure 8d‐ii).^[^ ^57^ ^]^ The scientists operated their device at low temperatures ($T_{e}=1100$ K, $T_{c}=640$ K) and achieved an output power density of P=11.5 mWcm^−2^ with a core efficiency of $\eta =3.9\times 10^{-3}$%, noting that the low thermal resistance of the columns (an area average of roughly $\varrho_{s}\approx 3.5$ cm^2^K W^−1^) resulted in large conductive heat transfer losses. Similarly, Bellucci et al.^[^ ^60^ ^]^ fabricated zirconia columns on the surface of a GaAs collector to separate it from a tungsten emitter by a distance of d=3 µm. This prototype produced a low open‐circuit current density of J≈30 nAcm^−2^ (peak power density P≈16 nWcm^−2^) despite its high temperature difference ($T_{e}-T_{c}=1468-523$ K) due to its large electrode work functions ($\phi_{e}\approx 4.5$ eV and $\phi_{c}\approx 3.46$ eV); however, it represented progress toward achieving ultra‐thin gaps in practical devices.

**Figure 9:**
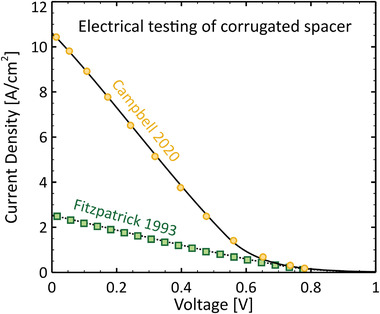
Example current–voltage measurement for a micron‐scale ceramic spacer film placed between two 1.27‐cm diameter electrodes with an emitter temperature of $T_{e}\approx 1300$ K (data from Campbell et al.^[^ ^48^ ^]^). The high current density indicates that ceramic spacer films are a path toward commercialization of TECs. Prior to this experiment, the nominal gap distance was measured using a capacitance technique to be d=3.4 µm; the difference from the nominal spacer height (2.3 µm) is attributable to factors such as electrode non‐parallelism, non‐uniform spacer placement, or the spacer folding under itself. A comparison is presented with a current–voltage measurement obtained from Fitzpatrick et al.^[^ ^46^ ^]^ that featured similar electrode temperatures and work functions but a larger gap distance (d=9.5 µm), highlighting the increase in current obtainable using ultra‐small electrode spacings. Finally, the black solid and dotted lines show predictions calculated using standard models, including space charge effects using best‐fit gap distances of d=3.7 µm and d = 9.5 µm, respectively.^[^ ^13^, ^37^ ^]^ The good agreement between the experimental results and the calculations reflects the efficacy of the models.

In contrast to these integrated supports that were permanently attached to the electrode substrate, Nicaise et al.^[^ ^190^ ^]^ and Campbell et al.^[^ ^48^ ^]^ developed free‐standing corrugated ceramic spacer films whose thermal resistance values were $\varrho_{s}=40-200$ cm^2^K W^−1^ (Figure 8d‐iii). These films, whose thicknesses were roughly 400 nm, consisted of hexagonally‐patterned U‐shaped channels with raised protrusions at the hexagon intersections that limited the area available for conductive heat transfer and established overall gap heights of d=2−8 µm. The spacers were manufacturable using standard microfabrication processes and were shown to be mechanically robust, such that they could be produced individually and then subsequently compressed between electrodes in TECs to establish temperature differences of several hundred kelvins while only permitting a few watts of heat flow.^[^ ^48^, ^190^ ^]^ In one demonstration, a 2.3 µm tall alumina‐hafnia spacer was placed between two 1.27‐cm diameter molybdenum electrodes with an emitter temperature of $T_{e}\approx 1300$ K, producing a peak power density of P≈1.5 Wcm^−2^ (including ideal resistive lead losses from $R_{\text{lead}}=4$ mΩ, see Equation (5) and **Figure** **9**).^[^ ^48^ ^]^ The ability of dielectric spacer films to sustain high temperatures and support high temperature gradients offers the promise of mass‐producible, high‐efficiency, high power output TECs.

### 5.5 Summary of Space Charge Mitigation Methods

To summarize, the particular strategy to mitigate space charge in a specific device depends on its performance targets, desired application, and manufacturing constraints. For example, solid‐state TECs may be most applicable, relative to vacuum gap TECs, to waste heat recovery or refrigeration because their low barrier heights allow sufficient electron emission at relatively low temperatures (however, we note that solid‐state TECs face many of the same challenges as thermoelectric converters). Plasma‐based and gate‐assisted TECs can afford larger gap distances and therefore exhibit lower conductive losses, though they do have other associated energy penalties. Finally, micron‐gap TECs supported by intragap spacers have larger parasitic losses than non‐spacer‐based strategies but benefit from their simple design and higher current densities at very low gap distances.

## 6 Challenges and Recommendations

We turn our attention now to highlighting the challenges currently facing thermionic energy converters and to suggesting directions for future research in the field. In particular, we will discuss the importance of accurate and consistent efficiency metrics for benchmarking different designs, the need for new low‐work‐function/high‐Richardson‐constant electrode materials, the prospect of mass‐producible gap‐maintaining spacers for TECs, and the potential of alternative TEC architectures.

### 6.1 Efficiency Metrics

Standardized definitions of efficiency facilitate quantitative comparisons of different energy conversion approaches. Here we discuss three complementary metrics and offer recommendations for reporting TEC performance in future publications. First, an optimistic metric called the *electronic efficiency*, $\eta_{e}$, has been used in the literature.^[^ ^13^, ^113^ ^]^ This quantity is the ratio of the power density produced (P, Equation (5)) to the net heat flux from thermionically emitted electrons ($Q_{\text{therm}}$, Equation (7)):

(15) $$\eta_{e}=\frac{P}{Q_{\text{therm}}}\times 100\%.$$

This electronic efficiency shares similarities with metrics used in adjacent fields such as thermophotovoltaics and to some degree thermoelectrics, and thus could appear useful for comparison between these differing technologies. However, it can be seen that this efficiency metric inflates efficiency values at high voltages that feature impractically low output current (and thus power) because it focuses solely on the heat carried by electrons. While useful for estimating the specific effectiveness of converting thermionic heat flux to electricity, this quantity is not a realistic measure of the overall energy conversion efficiency for TECs, and we do not recommend its use.

In contrast, in Equation (6), introduced earlier, we described the more realistic *core efficiency*, $\eta =\frac{P}{Q_{\text{in}}}\times 100$%, based on the ratio of electric power density produced to heat flux delivered to the emitter. We stress that scientists reporting core efficiencies should explicitly detail how the terms in this equation have been obtained. For calculated efficiencies of theoretical TEC architectures, the loss in output power due to lead resistance should be included (Equation (5)) and, at a minimum, conduction, radiation, lead conductive losses, and Joule heating (Equations (1) and (8)–(11)) should be calculated. In addition, modelers may need to include heat transfer due to vapor within the gap (Equation (12)),^[^ ^13^, ^46^ ^]^ electron reflection from the collector,^[^ ^75^ ^]^ and, in ultra‐small‐gap devices, near‐field radiative heat losses.^[^ ^44^, ^63^, ^64^ ^]^ Neglecting to include all of these effects can yield artificially high or optimistic efficiency predictions.^[^ ^111^, ^122^, ^124^ ^]^ We note that the Carnot efficiency limit for perfect heat engines operating between hot and cold reservoir temperatures of $T_{\text{hot}}$ and $T_{\text{cold}}$ is

(16) $$\eta_{\text{Carnot}}=\left(1-\frac{T_{\text{cold}}}{T_{\text{hot}}}\right)\times 100\%$$

and that the efficiency of heat engines operating at their maximum power point^[^ ^193^ ^]^ is often on the order of

(17) $$\eta_{\text{mpp}}\approx \left(1-\sqrt{\frac{T_{\text{cold}}}{T_{\text{hot}}}}\right)\times 100\%.$$

These yield practical limits of $\eta_{\text{Carnot}}=60$% and $\eta_{\text{mpp}}=37$% for typical $T_{\text{hot}}=T_{e}=1500$ K and $T_{\text{cold}}=T_{c}=600$ K. For core efficiency values derived from experimental data, we recommend that authors specify whether the power density reflects lead losses and whether the input heat flux includes heat lost through radiation in all directions (i.e., not just toward the emitter) and through conduction to any external supporting components. In principle, all heat input and loss mechanisms can be calculated or estimated, allowing a zero‐sum energy conservation balance.

While the core efficiency (Equation (6)) is a useful metric, it neglects certain energy losses such as those associated with electrical conversion, control systems, and heat generation irreversibility in practical systems. Accounting for these losses allows energy conversion systems to be compared more readily, especially across different platforms (i.e., comparing TECs to thermoelectric or even gas turbine‐powered generators). To accomplish this, a *system efficiency*, $\eta_{s}$, defined as the ratio of the power delivered by a TEC system ${\tilde{P}}_{d}$ [W] to the total rate of energy consumption by the TEC system ${\tilde{E}}_{c}$ [W] (in these terms, the tilde accent (∼) denotes non‐area‐specific units), can be calculated:

(18) $$\eta_{s}=\frac{{\tilde{P}}_{d}}{{\tilde{E}}_{c}}\times 100\%.$$

The rate of energy consumed by the TEC system ${\tilde{E}}_{c}$ is architecture‐dependent; for combustion‐based systems, it involves the enthalpy of the fuel and oxidizer streams, whereas for solar‐concentrating systems, it involves the aggregate insolation on the total area of the solar farm. The power delivered is given by

(19) $${\tilde{P}}_{d}=\eta_{c}S_{e}P-{\tilde{P}}_{c}$$

in which $\eta_{c}$ is the conversion efficiency for power boosting and inversion (necessary to change electricity from low VDC to high VAC) and ${\tilde{P}}_{c}$ [W] is the power consumed by support components (recall that P [Wcm^−2^] is the power density produced (Equation (5)) and $S_{e}$ [cm^2^] is the electron‐emitting surface area of the emitter). Power consuming support components could include fans or pumps to cool the collector, control electronics for the TEC, mass flow controllers for gas flow in combustion‐based systems, and sun‐tracking equipment for concentrated solar‐based systems. Calculating system efficiency values, in addition to facilitating comparisons between energy conversion platforms, has the added benefit of highlighting areas for improvement. For instance, an analysis may reveal the need for low‐power cooling systems that can efficiently remove heat from the collector (e.g., liquid cooling,^[^ ^158^ ^]^ heat pipes,^[^ ^82^, ^194^, ^195^ ^]^ or radiation^[^ ^196^ ^]^) or for efficient burner designs that recuperate waste exhaust heat and deliver it to the emitter.^[^ ^197^, ^198^, ^199^ ^]^ In summary, the system efficiency incorporates energy loss pathways that are external to a TEC but nevertheless important for its operation, allowing for more meaningful comparisons between energy generation architectures.

Finally, we note that most laboratory‐scale TEC implementations include energy consumption requirements that would be eliminated in commercial‐grade installations. These include powering vacuum pumps (unnecessary in hermetically sealed devices), counter‐heating vacuum flanges (this could be eliminated through careful system design), and supplying excess heat to the emitter to overcome excessively‐large conductive and radiative losses from exposed outer surfaces (this could be minimized by using larger‐diameter emitters and insulation). We recommend that such losses not be included in efficiency estimations, because they result in unrealistically low predictions. However, we again stress that scientists need to describe in detail the methods used to calculate any reported efficiency values, including any such exclusions.

### 6.2 Electrode Materials

Thus far we have stressed that, for currently available electrode materials (see Figure 4 and Table 1), high emitter temperatures are required to achieve high TEC efficiencies because emission increases exponentially with temperature (Equation (2)), and, as illustrated in Figure 2, thermionic heat transfer only dominates other heat transfer forms at high temperatures. However, as suggested by the dashed lines in Figure 4, high emission fluxes can also be achieved even at more moderate temperatures using electrodes that have low work functions and high Richardson constants.

**Figure 10:**
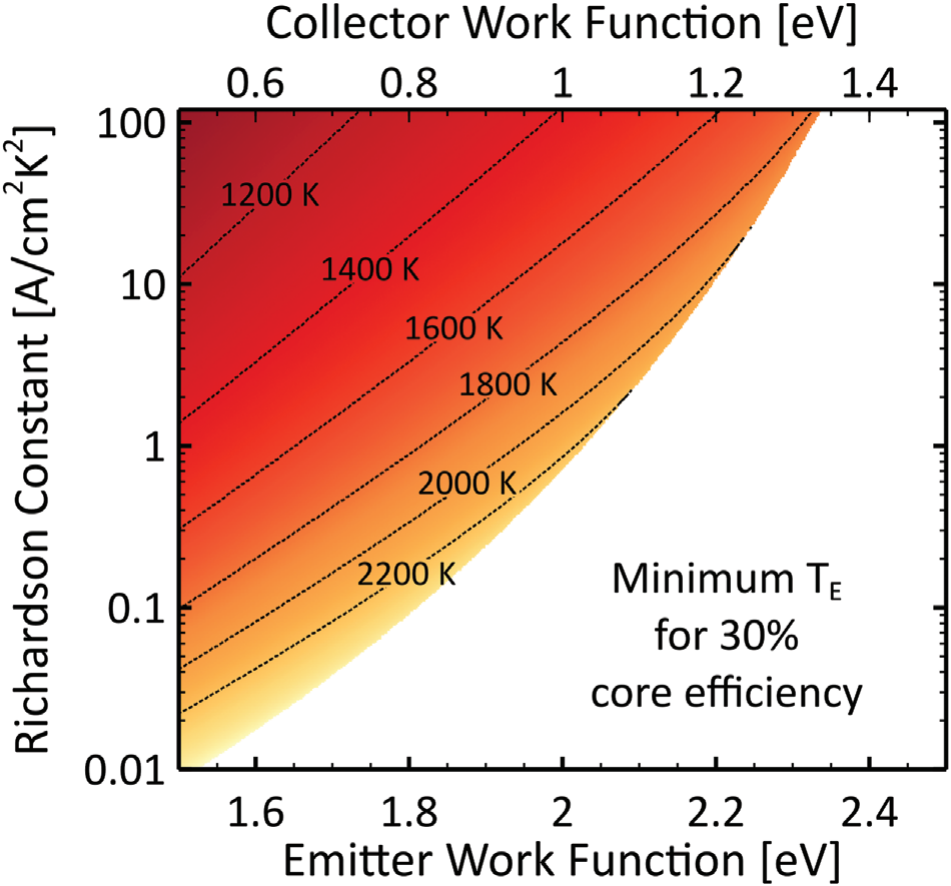
Contour plot showing the minimum emitter temperature $T_{e}$ required to achieve η=30% core efficiency as a function of the emitter work function $\phi_{e}$ and the emitter and collector Richardson constants $A_{e}=A_{c}$. Conditions: $\phi_{e}-\phi_{c}=1$ eV, $\varepsilon_{e}=0.2$, $\varepsilon_{c}=0.1$, $\varrho_{s}=100$ cm^2^K W^−1^, and $S_{e}=1$ cm^2^. To create this figure, for each work function‐Richardson constant combination, we iteratively determined the collector temperature $T_{c}$, output voltage $V_{o}$, and lead resistance $R_{\text{lead}}$ that maximized the core efficiency η while increasing the emitter temperature $T_{e}$ until a core efficiency of η=30% was obtained. We did not consider space charge effects, heat transfer due to cesium vapor, or structural heat transfer losses, and constrained the collector temperatures to $T_{c}\geq 300$ K. The upper limit of the ordinate corresponds to $A_{e}=A_{c}=120$ Acm^−2^K^−2^. Note that not all work function‐Richardson constant combinations depicted in this plot have been experimentally achieved; a survey of values demonstrated in the literature is provided in Figure 4 and Table 1.

To illustrate this, we calculated the minimum emitter temperature required to achieve a core efficiency of η=30% as a function of the emitter work function and Richardson constant (for a constant emitter‐collector work function difference of $\phi_{e}-\phi_{c}=1$ eV and $A_{e}=A_{c}$), as shown in **Figure** **10**. Moving right to left along the top of the graph, corresponding to the theoretical value of $A_{e}=A_{c}=120$ Acm^−2^K^‐^^2^, the required emitter temperature decreases by more than 600 K as the emitter work function decreases from about $\phi_{e}=2.3$ to 1.7 eV. The ability to achieve highly efficient operation at low temperatures could be enabled through electrodes with a combination of low work functions and high Richardson constants. Low temperature operation would beneficial because it could mitigate material thermal stability problems and further reduce parasitic condition through external support structures, thereby lowering TEC production costs and increasing overall system efficiencies. Figure 10 includes parasitic heat loss through conduction through interelectrode spacers, calculated according to Equation (11) using a thermal resistivity value of $\varrho_{s}=100$ cm^2^K W^−1^, which is similar to those reported recently in the literature.^[^ ^48^ ^]^ If conduction through electrode supports were suppressed completely, the temperatures of each of the contour lines shown would be reduced by roughly 200 K, that is, even lower emitter temperatures could still yield high core efficiencies. A combination of multiple strategies including electrode engineering and thermal enhancements will be required to achieve optimal performance in future TECs. In addition, we note that Figure 10 was constructed for the ideal case of negligible space charge effects, in order to highlight the importance of finding new low work function electrode materials. Were space charge included in these calculations, the required emitter temperatures shown would increase, and the magnitude of the increase would grow rapidly with the interelectrode gap distance d. Finally, Figure 10 shows that no practically sustainable emitter temperature is sufficiently high to render a core efficiency of η=30% for emitter work functions $\phi_{e}>2.4$ eV. Additional research is required to achieve optimal electrode properties for commercialization of thermionic converter technology. In Section 4, we discussed several methods by which electrode work functions can be reduced, including alkali metal or hydrogen coatings, electrostatic gating, and semiconductor effects. Given the large potential benefits in terms of power and efficiency of TECs, significant future research efforts should be devoted to these and other strategies that improve electron materials.

### 6.3 Interelectrode Supports to Maintain Small Gaps

As mentioned above, perhaps the foremost challenge facing TECs is overcoming the space charge effect. While strategies exist to circumvent this problem, such as introducing a cesium plasma to the gap^[^ ^14^ ^]^ or employing a third electrode,^[^ ^56^ ^]^ smaller vacuum gaps represent possibly the most straightforward solution. Methods to maintain microscale and nanoscale gaps by external means have included external silicon supports^[^ ^54^, ^55^, ^188^ ^]^ and micro‐manipulation systems,^[^ ^200^, ^201^ ^]^ and active gap control has also been accomplished using piezoelectric translators.^[^ ^83^ ^]^ Perhaps the most elegant solution, however, is to use fixed‐form inserts within the gap to define the electrode spacing. This approach has been implemented in several forms, including commercially available micron‐scale grains such as polystyrene particles,^[^ ^202^ ^]^ alumina beads,^[^ ^40^ ^]^ and silica spheres^[^ ^203^ ^]^; through lithographically defined insulators such as silicon dioxide or zirconia columns,^[^ ^57^, ^60^, ^204^, ^205^ ^]^ quartz standoffs,^[^ ^206^ ^]^ and photoresist pillars^[^ ^207^, ^208^ ^]^; and through freestanding structures such as corrugated ceramic films.^[^ ^48^, ^190^ ^]^

Critical parameters for fixed‐form spacers include their height, which determines the gap size, their thermal resistivity, which defines how much parasitic heat loss they allow, and their electrode shadowing (e.g., contact) area, which determines the cross section available for electrons and photons to pass between the electrodes through the gap. Spacers for practical and efficient large‐scale thermionic energy production must be mass producible and easily installable, mechanically robust, chemically and mechanically stable at high temperatures, and sufficiently thermally and electrically insulating.

**Figure 11:**
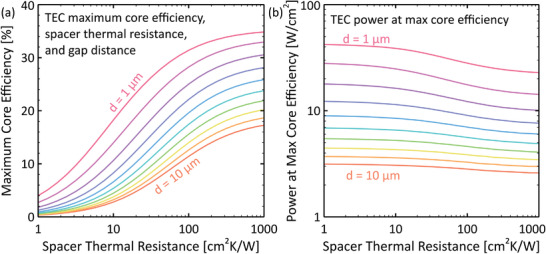
a) Maximum core efficiency of a micron‐gap TEC that uses intragap spacer supports (i.e., a spacer film) to separate its emitter and collector, as a function of the spacer supports' thermal resistance $\varrho_{s}$ [cm^2^K W^−1^], for integer gap distance values in the range d=1−10 µm. b) Corresponding power at point of maximum core efficiency. As the spacer resistance increases, the output voltage $V_{o}$ corresponding to maximum core efficiency decreases, resulting in a lower electron flux and a reduction in output power. Conditions: $T_{e}=1500$ K, $T_{c}=600$ K, $\phi_{e}=2.0$ eV, $\phi_{c}=1.0$ eV, $A_{e}=A_{c}=120$ Acm^−2^K^‐^^2^, and $S_{e}=1$ cm^2^. To create this figure, we modeled the emitter and collector as cesiated tungsten electrodes and calculated their emissivity using equations set forth in Fitzpatrick et al.,^[^ ^46^ ^]^ calculated the intragap electric field using equations available in Hatsopoulos and Gyftopoulos,^[^ ^13^, ^37^ ^]^ and included the small amount of heat transfer due to vapor within the gap.^[^ ^13^, ^46^ ^]^ Note that the highest spacer thermal resistance values within the domain of these graphs have not yet been achieved. However, values up to $\varrho_{s}\approx 200$ cm^2^K W^−1^have been experimentally demonstrated,^[^ ^48^ ^]^ and further enhancements will lead to added efficiency improvements.

Additional research is necessary to improve fixed‐form gap‐maintaining spacer supports. Most importantly, the thermal resistance of intragap supports must be increased in order to reduce parasitic conductive losses. The impact of these losses can be seen in **Figure** **11**a, which shows the maximum core efficiency of a micron‐gap TEC as a function of the thermal resistance of its interelectrode supports. Notably, the peak core efficiency decreases by an order of magnitude as the resistance decreases from $\varrho_{s}=1000$ to only $\varrho_{s}=1$ cm^2^K W^−1^, motivating research into materials and geometries that minimize conduction. Also, Figure 11a shows that the maximum TEC core efficiency increases as the gap spacing decreases and Figure 11b shows that the power increases with decreasing gap spacing as well.^[^ ^209^ ^]^ These improvements continue until nanoscale gap distances (d<0.5 µm, not shown) are reached, at which near‐field radiative heat transfer begins to increase.^[^ ^44^, ^63^, ^64^ ^]^ This motivates research into methods to produce thinner spacers (0.5<d<1.5 µm) that are still sufficiently robust to be mass‐produced and sustain compressive forces. Ultimately, thin, robust, insulating interelectrode supports, such as spacer films, will be beneficial for many TEC architectures, including traditional, PETE, and TIPV configurations.

### 6.4 Alternate Thermionic Configurations

In this review, we have considered several TEC designs, including traditional emitter‐collector pairs and more innovative designs involving heterostructure emitters, combined photovoltaic‐anode collectors, and third (gate) electrodes. By circumventing some of the setbacks of established TEC architectures, non‐traditional designs offer the possibility for increased robustness, alternative use cases, higher efficiency, and greater power production. Here we highlight two examples that we feel have particular potential, namely hybrid thermionic‐photovoltaic converters and gate‐assisted thermionic energy converters.

As introduced above, hybrid thermionic‐enhanced photovoltaic converters employ a combined electron collector‐photovoltaic receiver anode.^[^ ^59^ ^]^ Relative to traditional TECs, this is beneficial because it allows the hot emitter's infrared emission to generate current, and relative to TPVs, this is useful because the thermionically transferred electrons combine with holes produced in the photovoltaic cell, eliminating the need for an electron‐collection wire grid. If developed successfully, TIPV could be an expedient strategy at moderate emitter temperatures ($T_{e}\approx 1400$ K), for which the thermionic $Q_{\text{therm}}$ and radiative $Q_{\text{rad}}$ heat transfer fluxes are comparable (see Figure 2). As pointed out earlier, using low or even moderate emitter temperatures is advantageous because it reduces conductive losses to external components. Significant research is required to practically implement this technology. Perhaps the most important technical challenges are associated with the anode, which must have a top layer that can absorb electrons but is transparent to photons, must have a photovoltaic cell whose absorption is paired to the radiation emitted by the cathode, and must be able to withstand elevated temperatures to reduce the energy required for cooling.

Gate‐assisted thermionic energy converters are a modification of traditional TECs in which a third voltage‐biased gate electrode is placed between the emitter and collector in order to attract electrons and mitigate space charge effects.^[^ ^56^ ^]^ This configuration is advantageous because the lack of space charge permits greater gap distances, thereby reducing precision manufacturing costs and allowing more space for conductive insulation between the emitter and collector. Significant drawbacks of this approach include the fractional current loss to the gate electrode as well as the need for an aligned magnetic field to direct the electrons through the gate. Calculations have indicated that graphene gates, which have electron transparencies greater than 80%, may be feasible^[^ ^180^ ^]^; however, functional prototypes of such designs have not been reported yet.

Finally, we note that TECs need to be integrated into overarching heat transfer systems. In terms of energy input, they can, for instance, receive heat directly from primary processes (e.g., combustion, concentrated sunlight, or nuclear fission), use waste heat from industrial processes (e.g., metal foundries) or household appliances (e.g., hot water heaters or clothing dryers), or obtain heat stored in latent‐heat devices.^[^ ^3^, ^147^, ^182^ ^]^ In terms of heat rejection, TECs are well‐suited to serve as topping cycles that pass heat to other thermodynamic cycles, since TEC collectors often maintain elevated temperatures ($T_{c}\approx 600$ K). Bottoming cycles that receive heat from TECs could include thermoelectrics,^[^ ^210^, ^211^ ^]^ Stirling engines,^[^ ^212^ ^]^ or steam turbines.^[^ ^132^ ^]^ Beyond simply developing better thermionic architectures, additional research should be devoted to engineering systems that can optimally incorporate TECs.

## 7 Conclusion

Thermionic energy converters (TECs) use the spontaneous movement of electrons to generate electricity from heat using no moving parts. They offer key benefits such as versatility and scalability and can make use of numerous heat sources including combustion, concentrated sunlight, and radioactive isotope decay. Key challenges preventing the widespread use of TECs include parasitic heat transfer losses, the space charge effect, and problems associated with developing high‐performance electrode materials. The three primary methods demonstrated to overcome the space charge effect are introducing a cesium plasma into the interelectrode gap, employing a third voltage‐biased electrode to modify the intragap electric field, and reducing the gap distance (ideally to d<10 µm). A wide variety of electrode materials and configurations have been studied, including alkali metal‐coated substrates, hydrogen‐passivated diamond layers, carbon nanotubes, and suspended graphene sheets. Given the materials available today, significant power density output (P>1 Wcm^−2^) is limited to high‐temperature operation and is tied to reasonable electrical conversion efficiencies (η>10%), thereby requiring converters to be capable of handling heat input fluxes on the order $Q_{\text{in}}\approx 10$ Wcm^−2^.

Historical TEC prototypes were constructed through precision machining techniques, most of which were limited by their use of cesium plasmas and large gap distances to estimated efficiencies of η<15%. Recent efforts to improve the efficiency, power density, and versatility of TECs have resulted in novel electrode materials and designs as well as new methods to mitigate the space charge effect. In terms of electrodes, electrostatic gating can be used to reduce the Fermi level of graphene anodes and thereby reduce their work functions^[^ ^113^ ^]^; the surface photovoltage effect can be used to reduce the work function of *n*‐doped GaAs^[^ ^79^ ^]^; a heterostructure emitter can improve solar conversion efficiency in photon‐enhanced thermionic emission^[^ ^132^ ^]^; and photovoltaic cells can be incorporated into electron collectors in combined thermionic–photovoltaic converters.^[^ ^59^ ^]^ With regard to new methods to overcome space charge effects, voltage‐biased gates can be used to modify the intragap electric field^[^ ^56^ ^]^ and microfabricated corrugated thin ceramic films can be used to maintain micron‐scale electrode gap distances.^[^ ^190^ ^]^

Future publications should include descriptions of the efficiency metrics used for TEC evaluation; we recommend estimating both the core efficiency, $\eta =\frac{P}{Q_{\text{in}}}\times 100$%, which compares the output power density to the input heat flux (Equation (6)), and the system efficiency, $\eta_{s}=\frac{{\tilde{P}}_{d}}{{\tilde{E}}_{c}}\times 100$%, which compares the output power to the total energy consumed by the TEC and its supporting infrastructure (Equation (18)). Additional research efforts are needed to develop electrode materials that have low work functions and high Richardson constants, have low electronic resistance, and are tolerant of high temperatures. Research should continue toward the development of intragap supports (i.e., spacers^[^ ^190^ ^]^) that exhibit high thermal conductive resistance in order to produce mechanically robust TECs with ultrathin vacuum gaps. Finally, promising TEC architectures that deserve additional testing include hybrid thermionic‐photovoltaic converters, which harness energy from both electrons and photons produced by a hot emitter,^[^ ^59^ ^]^ and gate‐assisted thermionic energy converters, which use a third voltage‐biased electrode to suppress intragap space charge.^[^ ^56^ ^]^

## Conflict of Interest

The authors declare no conflict of interest.
